# Exploring the Inhibitory Mechanism of Approved Selective Norepinephrine Reuptake Inhibitors and Reboxetine Enantiomers by Molecular Dynamics Study

**DOI:** 10.1038/srep26883

**Published:** 2016-05-27

**Authors:** Guoxun Zheng, Weiwei Xue, Panpan Wang, Fengyuan Yang, Bo Li, Xiaofeng Li, Yinghong Li, Xiaojun Yao, Feng Zhu

**Affiliations:** 1 Innovative Drug Research and Bioinformatics Group, Innovative Drug Research Centre and School of Pharmaceutical Sciences, Chongqing University, Chongqing 401331, China; 2 State Key Laboratory of Applied Organic Chemistry and Department of Chemistry, Lanzhou University, Lanzhou 730000, China

## Abstract

Selective norepinephrine reuptake inhibitors (sNRIs) provide an effective class of approved antipsychotics, whose inhibitory mechanism could facilitate the discovery of privileged scaffolds with enhanced drug efficacy. However, the crystal structure of human norepinephrine transporter (hNET) has not been determined yet and the inhibitory mechanism of sNRIs remains elusive. In this work, multiple computational methods were integrated to explore the inhibitory mechanism of approved sNRIs (atomoxetine, maprotiline, reboxetine and viloxazine), and 3 lines of evidences were provided to verify the calculation results. Consequently, a binding mode defined by interactions between three chemical moieties in sNRIs and eleven residues in hNET was identified as shared by approved sNRIs. In the meantime, binding modes of reboxetine’s enantiomers with hNET were compared. 6 key residues favoring the binding of (S, S)-reboxetine over that of (R, R)-reboxetine were discovered. This is the first study reporting that those 11 residues are the common determinants for the binding of approved sNRIs. The identified binding mode shed light on the inhibitory mechanism of approved sNRIs, which could help identify novel scaffolds with improved drug efficacy.

Norepinephrine reuptake inhibitors (NRIs) are psychostimulant which is commonly used for mood and behavioral disorders^1^. Typical NRIs include the selective norepinephrine reuptake inhibitors (sNRIs)^2^, serotonin-norepinephrine reuptake inhibitor^3^ and others^4^. Currently, 4 sNRIs (atomoxetine, maprotiline, reboxetine and viloxazine) have been approved and marketed by either the U. S. Food and Drug Administration (FDA) or the European Medicines Agency for treating attention deficit hyperactivity disorder^5^ and depression^6^ (Fig. 1). Amongst these 4 sNRIs, reboxetine is a racemic mixture of (R, R)- and (S, S)- enantiomers. (S, S)-reboxetine showed 130-fold higher affinity to hNET than (R, R)-reboxetine, and was reported as the predominant influence on reboxetine’s steady state pharmacological property^7^. Due to the existing deficiencies of currently marketed sNRIs (their delayed onset of action^8^ and non- or partial-response^9^), new strategies were applied to enhance drug efficacy by improving their metabolic and pharmacological properties^10^,^11^ or by developing dual- and triple-acting antidepressants^12^. The binding mode shared by all approved and marketed sNRIs could contribute to the discovery of drug-like scaffold with enhanced efficacy^13^,^14^.

Human norepinephrine transporter (hNET), the drug target of sNRIs^15^, was reported to be closely relevant to various mood and behavioral disorders^16^,^17^ by facilitating the reuptake of norepinephrine from the synaptic cleft. Current understanding of hNET was based on the X-ray crystal structures of bacterial and invertebrate homologs, including the bacterial leucine transporter LeuT^18^,^19^,^20^,^21^, the *bacillus* neurotransmitter/sodium symporter MhsT^22^ and the *drosophila* dopamine transporter (dDAT)^23^,^24^. As the most recently determined template, dDAT’s X-ray crystal structure of high resolution revealed the binding of sNRIs (reboxetine and nisoxetine)^23^ and tricyclic antidepressant (nortriptyline)^24^. These co-crystallized structures showed a competitive binding of inhibitors to the S1 binding site by locking hNET in an outward-open conformation^23^,^24^. As shown in SI, Fig. S1, dDAT demonstrated the highest sequence identity among those hNET’s homologs, making it a new platform for constructing reliable models of sNRIs’ binding in hNET.

Many mutational and biomedical studies have been conducted to clarify the binding mode of sNRIs with hNET and identify key residues defining their recognition^25^,^26^,^27^. It was found that residue Asp75 in hNET was crucial for the interaction between sNRIs and hNET^27^. Moreover, 2 residues (Phe323 and Ser419) were identified as sensitive (with ≥5 fold-change in the loss- or gain-of-potency) to 3 sNRIs (atomoxetine, nisoxetine and maprotiline)^25^. Based on the X-ray crystal structure of hNET’s bacterial and invertebrate homologs^18^,^23^, 7 residues (Phe72, Asp75, Val148, Tyr152, Phe317, Phe323, Ser420) were also suggested as critical for some sNRIs (e.g. reboxetine) by visualizing the interaction distance between ligands and the target^23^. In the meantime, computational methods have been proposed and frequently used to elaborate the binding mode between sNRIs and hNET with great efficiency and accuracy^28^. These methods were applied (1) to elucidate binding mechanisms of substrates and inhibitors to monoamine transporter (MAT)^29^,^30^,^31^,^32^,^33^,^34^,^35^,^36^ (2) to discover novel scaffolds of MAT inhibitors by virtual screening^37^,^38^,^39^, and (3) to distinguish various molecular mechanisms of enantiomers binding to MAT^40^,^41^. As one of these powerful computational methods, the molecular dynamics (MD) providing atomic description of protein dynamics and flexibility^42^,^43^,^44^,^45^ was employed to simulate the large scale motions of MAT^27^,^46^,^47^. However, MD simulation has not yet been carried out to explore the binding of sNRIs to hNET. Moreover, the variation on binding modes behind the affinity discrepancy of reboxetine’s enantiomers remains elusive. Thus, there is an urgent need to reveal the mechanism underlying sNRIs’ pharmacodynamics and target recognition^23^,^25^.

In this work, multiple computational methods were integrated to explore the inhibitory mechanism of approved sNRIs (atomoxetine, maprotiline, reboxetine and viloxazine). First, a recently reported co-crystal structure of *drosophila* dopamine transporter (dDAT) in complex with reboxetine^23^ was used as a template to construct the homology model of hNET. Then, 4 studied sNRIs were docked into hNET for MD simulation, and 3 lines of evidences were provided to verify the simulation results. Consequently, a binding mode shared by approved sNRIs was discovered by clustering the binding free energies of residues. Moreover, the binding modes of 2 reboxetine enantiomers with hNET were compared, and residues favoring the binding of (S, S)-reboxetine over that of (R, R)-reboxetine were discovered. The identified binding mode shed light on the inhibitory mechanism of approved sNRIs, which could help identify novel scaffolds with improved drug efficacy^13^.

## Materials and Methods

### Homology Modeling

The homology model of hNET was constructed by using the automated mode in *SWISS-MODEL*^48^, based on a recently determined dDAT’s X-ray crystal structure^23^ of 3.0 Å resolution (PDB entry: 4XNX from Arg25 to Pro596). As shown in SI, Fig. S1, dDAT^23^ demonstrated 61% sequence identity to hNET, which was much higher than that (23%) of LeuT^18^. The sequence coverage of the constructed hNET model was between Arg56 and Pro594 covering hNET’s all transmembrane (TM) regions and corresponding extracellular loops. To validate the constructed homology model, the Ramachandran plot in *PROCHECK*^49^ was further exploited. Finally, two functional Na^+^ in dDAT (PDB entry: 4XNX^23^) were added to their interacting sites in hNET by structural superimposition via *PyMOL*^50^.

### Molecular Docking

Initial binding conformations of sNRIs in hNET were obtained by molecular docking using *Glide*^31^ of standard precision. The docking grid box was defined by centering (R, R)-reboxetine (PDB entry: 4XNX^23^) in the modeled hNET using the *Receptor Grid Generation tool* in *Glide*. Docking poses of sNRIs, with the most similar conformations or orientations as (R, R)-reboxetine in dDAT^23^, were chosen for MD simulation. Furthermore, a cross-docking approach was applied in this work to validate the docking method. Detailed supporting information was provided in *SI, Methods*.

### System Setup of the Protein-Ligand and Membrane

The *Membrane Builder* in *CHARMM-GUI*^51^ was used to embed the sNRIs-hNET complexes into the explicit POPC lipid bilayer. TIP3P water^52^ was then positioned above and below the constructed bilayer (20 Å thickness). Na^+^ and Cl^−^ were added to keep the environmental salt concentration at 0.15 M. As a result, each periodic cell of the entire system (83 Å × 83 Å × 127 Å) contained ~96,400 atoms. Detailed supporting information was provided in *SI, Methods*.

### MD Simulation

*AMBER14*^53^ using GPU-accelerated *PMEMD* was applied to carry out MD simulation. The force field *ff14SB*^54^ was used for protein, and *Lipid14*^55^ were utilized for lipid. TIP3P water’s ions parameters were directly adopted from previous publication^56^. sNRIs’ parameters were generated using the *General AMBER force field*^57^ and the charges of sNRIs’ atoms were derived by the *Restrained Electrostatic Potential partial charges*^58^ in *Antechamber*^59^. *Gaussian09*^60^ was applied to optimize the geometry and calculate the electrostatic potential at the HF/6–31G* level. In all simulations, a sequential process was executed (minimization, heating and equilibration). After this, 150 ns production MD simulation at 310 K and 1 atm was conducted in NPT ensemble with the periodic boundary conditions. Meanwhile, the long-range electrostatic interactions (cutoff = 10.0 Å) was treated using Particle-Mesh Ewald method^61^. The *SHAKE algorithm*^62^ was applied to constrain the bond lengths involving bond to hydrogen atoms, and in simulation the integration time step was set as 2 fs. Supporting data were provided in the *SI, Methods*.

### Calculation of the Binding Free Energies

The energies of each sNRI binding to hNET (ΔG_MM/GBSA_) without the entropic effect were analyzed by the MM/GBSA approach with single-trajectory^63^,^64^. The last 50 ns of simulation (500 snapshots) was used for binding free energy calculation. For each snapshot, the energy of sNRI binding to hNET was computed by:


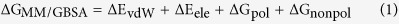


In Eq. (1), ΔE_vdW_ indicates the van der Waals energy, and ΔE_ele_ denotes the electrostatic energy. ΔG_pol_ is the polar solvent interaction energy calculated by solving the GB equation. ΔG_nonpol_ is the non-polar solvation contribution and estimated as 0.0072 × ΔSASA using the LCPO method^65^, where the SASA denotes the solvent accessible area. Supporting data were provided in the *SI, Methods*.

### Analysis of the Per-residue MM/GBSA Free Energy Decomposition

In order to analyze the per-residue contribution to sNRIs’ binding to hNET, energy was calculated by the approach of MM/GBSA decomposition using *mmpbsa.pl* plugin in *AMBER14*^53^. The per-residue energy(
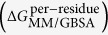
 without entropic effect could be computed by:


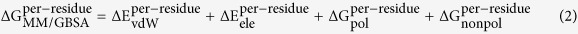


Most of the terms in Eq. (2) are defined in the same way as that in Eq. (1), but the energy of the non-polar solvent interaction 
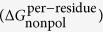
 was computed by recursively approximating a sphere around an atom from an icosahedron^53^. Supporting data were provided in the *SI, Methods*.

### Hierarchical Clustering of Residues Based on Their Energy Contributions

Energy contributions of certain residue to 4 approved sNRIs (atomoxetine, maprotiline, reboxetine and viloxazine) rendered a 4 dimensional vector. The clustering tree of residues contributing to at least one studied sNRI (≠0 kcal/mol) in hNET’s binding was constructed by the statistical analyzing package R^66^. The Manhattan distance was selected to calculate similarities among vectors:


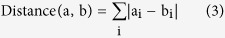


where i indicates the dimension of the residue’s energy contribution a and b. *Ward’s minimum variance module*^67^ in *R*^66^ for hierarchical clustering was adopted for minimizing the total variance within cluster. The hierarchical tree was depicted by the latest version of *iTOL*^68^. The residues favoring sNRI’s binding are colored in red (the one with the highest contribution was colored as red and the lower contribution one was set to fade gradually to white). In the meantime, the residues hampering sNRI’s binding are displayed in blue (the highest one was colored as blue and the lower one was set to fade gradually to white). The white color here denotes residue with no contribution to sNRIs’ binding.

## Results and Discussion

### Construction of the sNRIs-hNET Complexes

The homology model of the hNET was built on a 3.0 Å high-resolution crystal structure of the dDAT (PDB entry: 4XNX^23^). The sequence identity between the modeled fraction of hNET and dDAT was 61% (SI, Fig. S1). As shown in SI, Fig. S2, the modeled hNET contained the whole TM helices and the S1 binding site, and showed high degree of homology to dDAT’s crystal structure. Ramachandran plot^49^ showed a reasonable homology model, which identified 99.4% residues in the “allowed region” (SI, Fig. S3).

Then, the co-crystallized and the cross docking poses of 2 sNRIs (R, R)-reboxetine and nisoxetine (PDB entries: 4XNX and 4XUN^23^) were superimposed to assess the reliability of the docking methods applied in this work. As a result, these 2 poses were consensus with each other (SI, Fig. S4), indicating the correct initial binding poses generated here. 4 approved sNRIs (atomoxetine, maprotiline, reboxetine and viloxazine) and 2 standard sNRIs (nisoxetine and talopram) widely used in scientific research were included in this study for MD simulation to overcome the probable over-fitting result. Although no co-crystallized structures of atomoxetine, maprotiline, (S, S)-reboxetine, talopram or viloxazine was reported, their resulting docking poses resembled in orientation as the co-crystallized pose of (R, R)-reboxetine (SI, Fig. S5).

### Assessment of the Binding Mode of sNRIs-hNET

#### Evaluation of the Simulation Stability

The dynamic stabilities of the 6 systems (atomoxetine, maprotiline, nisoxetine, reboxetine, talopram and viloxazine) along MD simulation were measured by their average distance from the initial structures in terms of RMSD. The RMSD values of protein backbone atoms, ligand heavy atoms and binding site residue atoms (around 5 Å of ligand) in the entire MD simulation trajectories were illustrated in SI, Fig. S6. All 6 simulated systems reached equilibration state after 100 ns with only little fluctuation in the monitored RMSD.

#### Analysis of the Binding Free Energy

**Figure 2:**
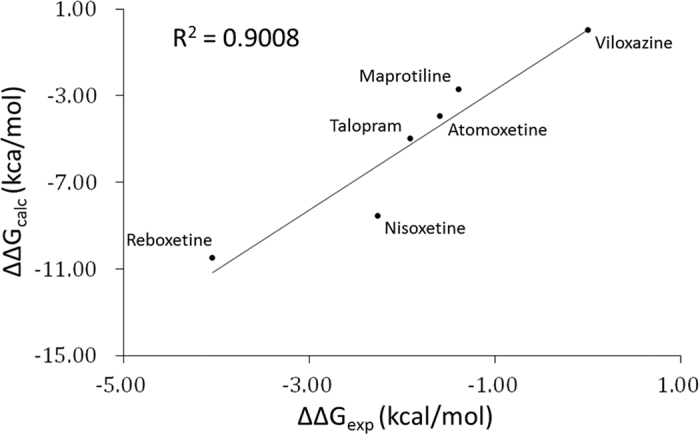
Correlation between energy difference of 6 studied sNRIs calculated in this work (ΔΔG_calc_) and that estimated based on experiments^7^,^27^,^69^,^70^,^71^ (ΔΔG_exp_).

The MM/GBSA calculations were performed to quantitatively analyze the binding free energies of hNET in complex with sNRIs (Table 1). The predicted binding free energy (ΔG_MM/GBSA_) for hNET with atomoxetine, maprotiline, nisoxetine, talopram, viloxazine and (S, S)-reboxetine were −41.42, −40.21, −46.05, −42.47, −37.48 and −47.67 kcal/mol, respectively. Meanwhile, ΔG_exp_ = RTln(K_i_) was used to convert the experimental K values^7^,^27^,^69^,^70^,^71^ to the binding free energies (ΔG_exp_). As shown in Table 1, binding free energies for each system were overestimated compared to the experimental results. If one is only interested in the relative order of binding affinities of structurally similar ligands of resemble binding modes, the entropy effects could be omitted^72^. Therefore, the energy differences (Table 1) calculated (ΔΔG_MM/GBSA_) and estimated by experimental data (ΔΔG_exp_) among 4 sNRIs could tell whether these overestimations were originated from the omission of entropy effects. ΔΔG_MM/GBSA_ was shown in Fig. 2 to correlate well with ΔΔG_exp_ (R^2^ = 0.9008). In spite of an overestimation of ΔG_MM/GBSA_ comparing to the ΔG_exp_^7^,^69^,^70^, the ascending trend of ΔG_exp_ was reproduced well by ΔG_MM/GBSA_. The overestimated energy in our study was in agreement with the reported over-evaluation using the approach of MM/GBSA^73^,^74^,^75^. Each component of energy in Eq. (1) was illustrated in SI, Table S1. In particular, ΔE_vdW_ and ΔE_ele_ mainly contributed to the binding of sNRIs with hNET, while the polar solvent energy (ΔG_pol_) impeded the binding.

#### Verifying the Resulting Models of MD Simulation

Besides the good correlation between the results of simulation and experiments in previous section, 3 lines of evidence further verified our resulting model of MD simulation. The first line of evidence was from the recently identified key residues that control sNRIs’ selectivity in hNET^76^. As reported, a mutational analysis of 6 diverging residues (Ala145, Tyr151, Ile315, Phe316, Ser420 and Ala426) in the central binding site (S1 site) of hNET to the complementary residues in the human dopamine transporter (hDAT) transferred a hDAT-like pharmacology to hNET, showing that those 6 residues were collectively key residues for sNRIs’ selectivity^76^. To investigate the influence on the binding of 6 studied sNRIs to the hDAT-like hNET^76^, *in silico* mutations on these 6 residues in hNET to the identity of the corresponding residues in hDAT (A145S-Y151F-I315V-F316C-S420A-A426S) were conducted in this work. As illustrated in SI, Fig. S7, 6 studied sNRIs (atomoxetine, maprotiline, nisoxetine, talopram, viloxazine and (S, S)-reboxetine) in complex with the hDAT-like hNET were analyzed by adding 20 ns simulation to the models of the wild type hNET constructed in this work. The resulting binding free energy of 6 sNRIs was calculated, and the fold-changes in their binding affinity induced by hDAT-like hNET mutations from Andersen’s experimental study^76^ were listed in Table 2 (detail information of each energy term can also be found in SI, Table S3). As shown, the fold-changes in binding affinities of 4 sNRIs (atomoxetine, nisoxetine, reboxetine and talopram) reported in Andersen’s study^76^ were reproduced well by our calculated ΔΔG_MM/GBSA_ (ΔΔG_calc_). In particular, the ΔΔG_calc_ were 0.47, 1.15. 2.89 and 3.70 kcal/mol for reboxetine, atomoxetine, nisoxetine and talopram, respectively, which were comparable and followed the same trends as the reported increases in binding free energies (ΔΔG_exp_) induced by hDAT-like hNET mutations (1.38, 1.97, 3.04 and 3.16 kcal/mol for reboxetine, atomoxetine, nisoxetine and talopram)^76^. Since those additional 20 ns simulations were all based on the resulting hNET models constructed in this work, this reproduction of experiments by simulation could act as one line of evidence for verifying our resulting simulation models. In the meantime, the binding free energies of 2 sNRIs (maprotiline and viloxazine) not included in Andersen’s study^76^ were also calculated and listed in Table 2 and SI, Table S3. The bindings of both sNRIs were affected significantly by hDAT-like hNET mutations. These results supported Andersen’s findings that 6 residues in the S1 site of hNET controlled sNRIs’ selectivity^76^. SI, Fig. S8 illustrated changes in the conformation and orientation of both hNET’s binding site and those 6 studied sNRIs within it.

The second line of evidence, coming from the reported mutagenesis experiments on the sensitivity profiles of hNET’s residues, indicated that our resulting models of MD simulation were capable of distinguishing sensitive residues from non-sensitive ones. As reported, the sensitivity profiles could shed light on the binding mode of sNRIs^25^. The residues’ sensitivity to the binding of sNRIs’ could be estimated by the variation in the binding free energy before and after the *in silico* mutation. In this work, 2 sensitive mutations (S419T and F323Y with ≥5-fold changes in binding affinity) and 2 non-sensitive mutations (F72Y and N153S without markedly change in potency) of 2 sNRIs (atomoxetine and maprotiline) identified by previous experiments^25^ were selected, and their sensitivities were explored by *in silico* mutation study. Particularly, hNET of single-point mutations (S419T, F323Y, F72Y and N153S) in complex with those 2 sNRIs were analyzed by additional 20 ns simulation based on the models of the wild type hNET constructed in this work (SI, Fig. S9). The calculated binding free energies of 2 sNRIs and the fold-changes in their binding affinity from Sorensen’s experiments^25^ induced by those single-point mutations were shown in Table 3, and information of each energy term was listed in SI, Table S4. As shown, the sensitivity profiles of 4 mutations reported in Sorensen’s work^25^ were successfully discovered by the calculated ΔΔG_cal_ in this work. Particularly, our simulation discovered F72Y and N153S as non-sensitive mutations to both sNRIs. In Table 3, the ΔΔG_calc_ were between −0.31 and 0.05 kcal/mol. The corresponding range of fold-changes in potency (FC_potency_) could thus be estimated as from 0.59 to 1.09 by the equation ΔΔG_calc_ = RTln (FC_potency_), which were comparable to the experimentally estimated non-sensitive FC_potency_ (from 0.59 to 2.34)^25^. Meanwhile, S419T and F323Y were identified as sensitive to both sNRIs. Their ΔΔG_calc_ were between 0.96 and 1.93 kcal/mol. The corresponding range of FC_potency_ were estimated as from 5.05 to 25.95, which were also comparable to those experimentally estimated sensitive FC_potency_ (from 3.89 to 12.67)^25^. The distinct difference in FC_potency_ between sensitive and non-sensitive mutations indicated that our resulting models were capable of distinguishing the sensitive mutations (S419T and F323Y) from the non-sensitive ones (F72Y and N153S). As those *in silico* mutational studies were based on models constructed in this work, their ability to identify the sensitivity profiles of hNET’s residues could be considered as another line of evidence to verify our resulting models. SI, Fig. S10 illustrated the changes in the conformation and orientation of both hNET’s binding site and 2 sNRIs within it.

**Figure 3:**
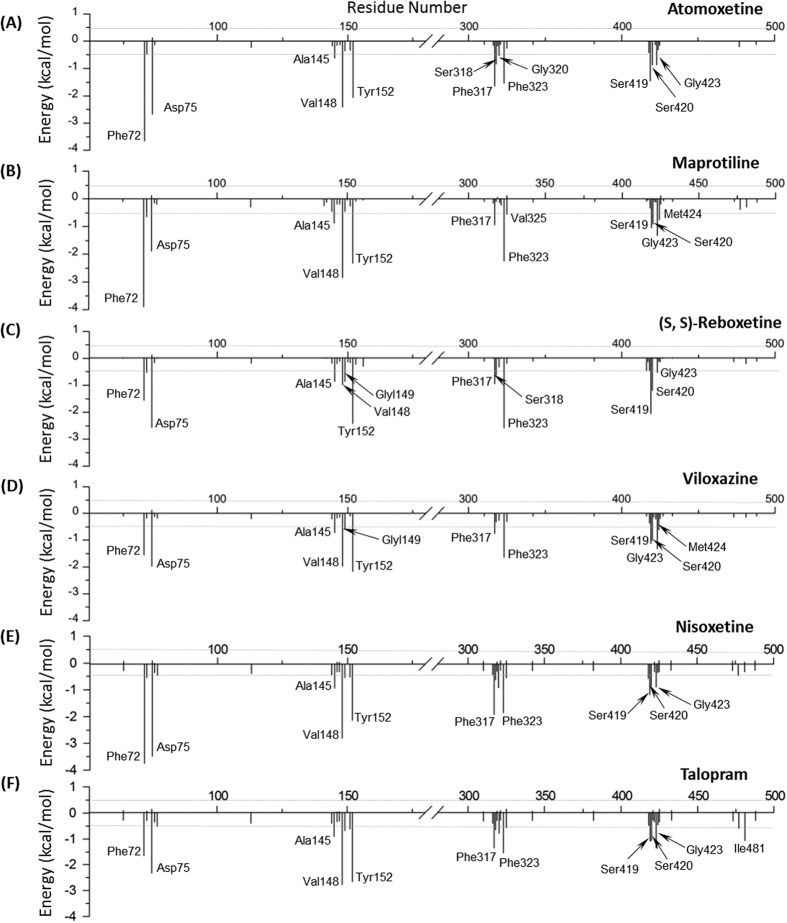
Per-residue binding free energy decomposition of 6 studied sNRIs-hNET complexes. Residues with high energy contribution (the absolute energy contribution ≥0.5 kcal/mol) were labeled.

The above evidence was supported further by the third line of evidence from the crystallography study, which reported co-crystalized structures of ligands (nisoxetine and reboxetine) with dDAT^23^. Based on their structures, these 2 sNRIs appeared to have very similar modes of binding and action. Of these 2 complexes, the amino groups of both ligands interact with the residues Phe43 and Asp46 (the corresponding residues Phe72 and Asp75 in hNET)^23^. Moreover, the cavity formed by residues Phe43, Ala44, Phe319 and Ser320 (the corresponding residues Phe72, Ala73, Phe317 and Ser318 in hNET) was occupied by the amine group of nisoxetine and the morpholine nitrogen of reboxetine^23^. In addition, two aromatic rings of both sNRIs were involved in the hydrophobic cleft bordered by residues Val120, Tyr123, Tyr124, Phe319, Phe325 and Ser422 (the corresponding residues Val148, Tyr151, Tyr152, Phe317, Phe323 and Ser420 in hNET), which further stabilized both sNRIs in the S1 binding site^23^. In this study, all of those residues contributed significantly (≥0.5 kcal/mol) to the binding of sNRIs (illustrated in Fig. 3), which could be the third line of evidence verifying our resulting simulation models.

#### Analysis of the sNRIs’ Binding Mode in hNET

The representative structures of 6 sNRIs extracted from the equilibrated simulation trajectory slightly shifted in conformation comparing to their corresponding docking poses (SI, Fig. S11), and key interactions between the ligands and Asp75 of hNET were retained. Moreover, per-residue free energy decomposition could help identify key residues in the binding of sNRIs to hNET. As shown in Fig. 3, 12, 13, 10, 11, 10 and 12 residues were recognized as high contribution ones (≥0.5 kcal/mol) for the binding of atomoxetine, maprotiline, nisoxetine, talopram, viloxazine and (S, S)-reboxetine, respectively. On one hand, it is clear to see that energies of different residues to the same sNRI differ significantly (from −0.53 kcal/mol for Gly320 to −3.67 kcal/mol for Phe72 in atomoxetine’s binding), and energies of the same residue to different sNRIs also vary greatly (the contributions of Asp75 are from −1.97 kcal/mol for viloxazine to −3.29 kcal/mol for nisoxetine). On the other hand, Fig. 3 also reflects similarity among sNRIs to some extent. As reported, the shared binding mode of approved sNRIs are very helpful in discovering hits or lead compounds with improved efficacy^13^,^14^, which inspires us to further explore the binding mode shared by approved sNRIs.

### Identification of the Shared Binding Mode of Approved sNRIs

**Figure 4:**
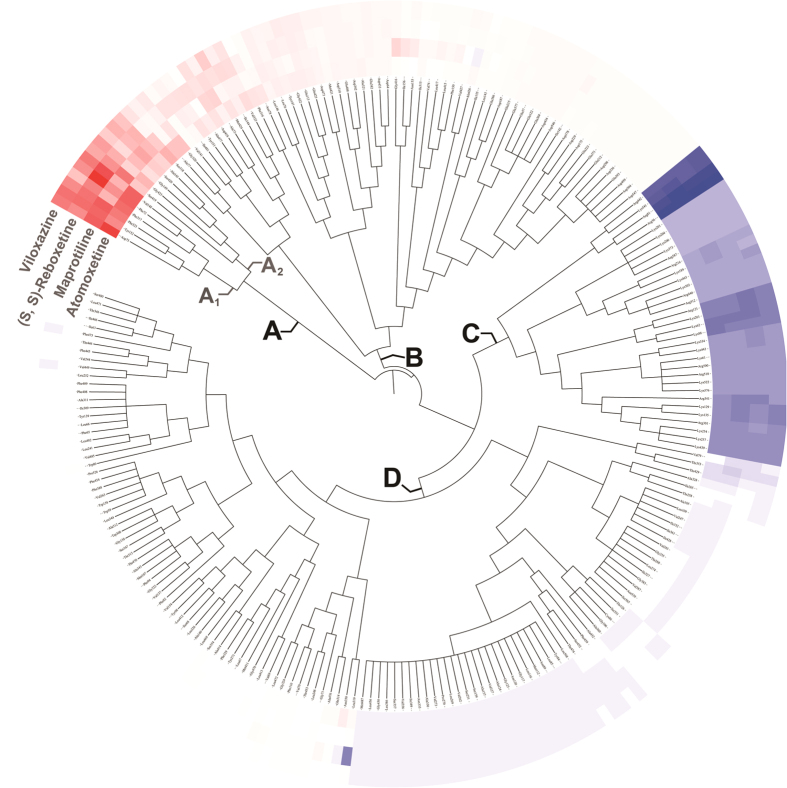
Tree of 238 residues with contribution to at least one studied sNRI in binding hNET by hierarchically clustering their energies. The residues favoring sNRI’s binding are colored in red (the one with the highest contribution was colored as standard red and the lower contribution one was set to fade gradually to white). In the meantime, the residues hampering sNRI’s binding are displayed in blue (the highest one was colored as standard blue and the lower one was set to fade gradually to white). The white color here denotes residue with no contribution to sNRIs’ binding.

Among those 538 residues in hNET, 238 were with energy contribution to at least 1 approved sNRI. To characterize the most favorable binding mode shared by approved sNRIs, hierarchical clustering with ward algorithm^67^ was exploited to identify hot spots from those 238 residues based on their energies. In Fig. 4, 4 groups of residues (A, B, C and D) were discovered. The residues favoring sNRI’s binding were colored in red. The residue with the highest contribution (−3.91 kcal/mol) was colored as standard red. The color of the lower contribution one was set to fade gradually towards white (no contribution). In the meantime, the residues hampering sNRI’s binding were displayed in blue. The highest one was colored as standard blue (0.20 kcal/mol) and the color of the lower one was set to fade gradually towards white. Importantly, it should be noticed that the highest energy contribution of residue favoring sNRI’s binding is much larger (about 19 times) than that hampering the binding.

As shown in Fig. 4, energy contribution of group A (Phe72, Asp75, Ala145, Val148, Gly149, Tyr152, Phe317, Phe323, Ser419, Ser420, Gly423) were consistently higher across all approved sNRIs than that of group B, C and D, indicating a crucial role played by group A in sNRIs’ binding. In particular, the sum of group A’s energy contributions accounted for the major part of the total energy (77.80% for atomoxetine, 78.06% for maprotiline, 78.95% for (S, S)-reboxetine and 75.27% for viloxazine). Those 11 residues revealed a similar pattern in drug binding in spite of their distant chemical scaffolds, and therefore were identified as hot spots for sNRIs’ binding. Moreover, residues in subgroup A_1_ (Phe72, Asp75, Val148, Tyr152, Phe317 and Phe323) contributed much higher to sNRIs’ binding than those in subgroup A_2_ (Ala145, Gly149, Ser419, Ser420 and Gly423). The sum of subgroup A_1_’s energy contributions constituted 60.15%, 58.87%, 53.54% and 50.27% of the total energies for atomoxetine, maprotiline (S, S)-reboxetine and viloxazine, respectively.

**Figure 5:**
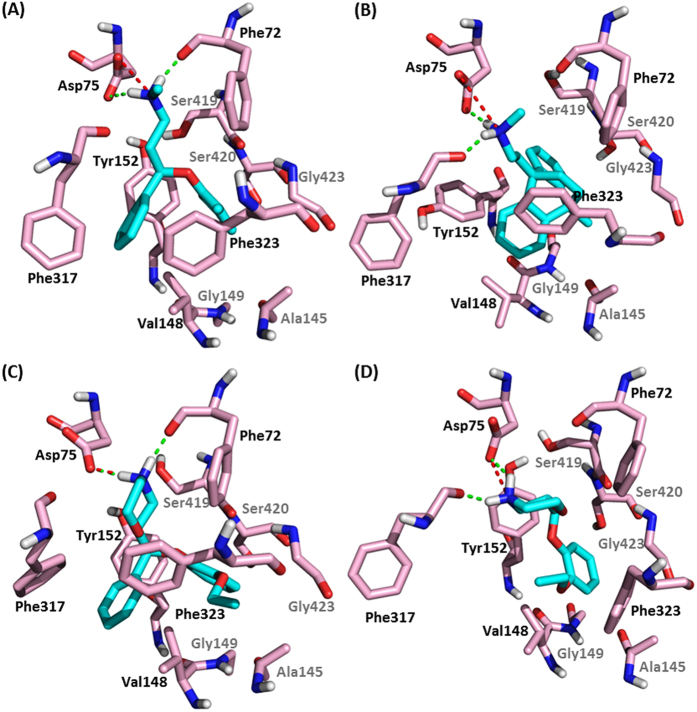
Binding modes of approved sNRIs (**A**) atomoxetine (**B**) maprotiline (**C**) (S, S)-reboxetine and (**D**) viloxazine with hNET identified in this work. The ligands were displayed in cyan and hot spot residues were depicted in light pink. The salt bridge interaction was displayed in red dashed line and the hydrogen bond interaction was in green. Hot spot residues in subgroup A_1_ (strong interaction) and A_2_ (relatively strong interaction) shown in Fig. 5 were labeled in black and gray, respectively.

**Figure 6:**
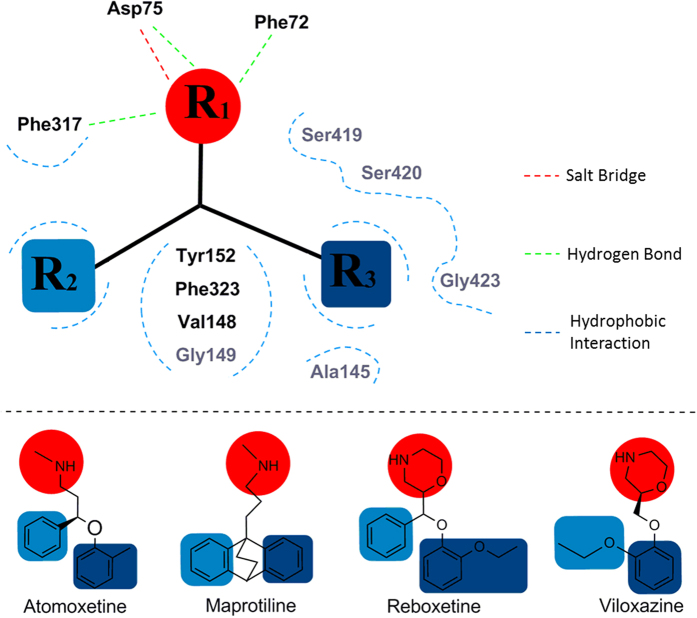
The binding mode shared by approved sNRIs with hNET. The identified salt bridges, hydrogen bonds and hydrophobic interactions were depicted in red, green and blue dashed lines, respectively. The red color (R_1_) indicated the chemical moiety with salt bridge and hydrogen bond interaction with residues in the vicinity, while the light blue (R_2_) and dark blue (R_3_) represented the chemical moiety with only hydrophobic interaction with its nearby residues. Each chemical moiety was generalized by the superimposition of 4 sNRIs in the S1 pocket. The residues in dark (Phe72, Asp75, Val148, Tyr152, Phe317, and Phe323) belonged to the subgroup A_1_ (Fig. 5), and the residues in gray (Ala145, Gly149, Ser419, Ser420 and Gly423) were clustered into subgroup A_2_ (Fig. 5).

As shown in Fig. 5, the amino group of all 4 sNRIs pointed to the residue Asp75, and the rest of hot spot residues formed the hydrophobic part of the binding pockets. To measure the conformational shift among the binding pockets of 4 sNRIs, the RMSDs of the 11 hot spot residues were calculated. The resulting RMSDs of different sNRIs’ binding pockets were all <3.0 Å with the highest one of 2.8 Å and the lowest one of 1.7 Å. In addition, in order to compare the orientations of these sNRIs, the superimposition of all sNRIs were shown in SI, Fig. S12, which gave a resembled orientation among them. Thus, the generalized binding mode of sNRIs with hNET was schematically displayed in Fig. 6. As illustrated, the binding mode was represented by the interactions of salt bridge, hydrogen bond and hydrophobic contact between three chemical moieties and eleven hot spot residues (Phe72, Asp75, Ala145, Val148, Gly149, Tyr152, Phe317, Phe323, Ser419, Ser420, Gly423). In Fig. 6, these three chemical moieties were illustrated by the color of red (R_1_), light blue (R_2_) and blue (R_3_). Residues with strong (subgroup A_1_) energies were marked in black, and residues with relatively strong (subgroup A_2_) energies were shown in gray (Figs 5 and 6). Particularly, the moiety R_1_ mainly engaged in the formation of salt bridge interaction and the hydrogen bond with Asp75 and Phe72 or Phe317; R_2_ formed hydrophobic interactions with Val148, Gly149, Tyr152 and Phe323 and also contacted with Phe317; R_3_ contacted hydrophobically with Ala145, Ser419, Ser420 and Gly423.

Among those 11 identified hot spot residues, 5 (Phe72, Val148, Gly149, Phe323 and Ser419) were studied in Sorensen’s work^25^. By measuring the changes on the inhibitory potencies before and after the mutation, Sorensen *et al*. found 2 sensitive mutations (S419T and F323Y, ≥5 -fold changes for atomoxetine and maprotiline) and 2 mutations (F72Y and N153S) without markedly decrease in potency of both sNRIs. Moreover, recently determined co-crystallized structures of sNRIs in dDAT (a homologous structure of hNET) could shade light on their binding mechanism. However, besides (R, R)-reboxetine and nisoxetine^23^, no structure of any sNRIs complexed with hNET or its homologous structure was reported. According to (R, R)-reboxetine’s co-crystallized structure^23^, 7 residues (Phe72, Asp75, Val148, Tyr152, Phe317, Phe323, Ser420) out of those 11 hot spots were suggested as critical residues by visualizing interaction distances between ligand and dDAT^23^. In addition, crystal structures of LeuBAT (engineered LeuT) in complex with 4 selective serotonin reuptake inhibitors (SSRIs), 2 serotonin–norepinephrine reuptake inhibitors (SNRIs) and 1 tricyclic antidepressant (TCA) were determined and reported to harbor a human monoamine transporter-like pharmacology^77^. Analysis of these SSRIs, SNRIs and TCA ligands complexed with LeuBAT helped to identify the binding pocket defined by Tyr21, Asp24, Val104, Ala105, Tyr108, Phe253, Gly256, Phe259, Ser355, Gly359, Asp404 and Thr408 (corresponding residues in hNET were Phe72, Asp75, Val148, Gly149, Tyr151, Phe317, Gly320, Phe323, Ser419, Gly423, Asp473 and Ala477). Among these corresponding residues, 8 (Phe72, Asp75, Val148, Gly149, Phe317, Phe323, Ser419, Gly423) were overlapped with those 11 hot spots identified by this study. Overall, these 11 residues were reported for the first time as the common determinants for the binding of all approved sNRIs.

Further analysis on energy contributions of sNRIs’ different chemical moieties reveals a vital role of chemical moiety R_1_ in sNRIs-hNET recognition^25^. R_1_ forms salt bridge with Asp75 and hydrogen bonds with Asp75, Phe72 and Phe317, which in total consists of 34.29%, 23.04%, 28.04% and 22.70% of binding free energies for atomoxetine, maprotiline, (S, S)-reboxetine and viloxazine, respectively. To further understand these interactions anchoring different sNRIs into the binding site, salt bridge and hydrogen bond were monitored along the entire MD simulation. Detail information can be found in *SI, Results and Discussion*.

### Two Reboxetine Enantiomers Distinguished by Their Binding Modes

(S, S)-reboxetine showed 130-fold higher affinity to hNET than its (R, R) enantiomer, and was reported as the predominant influence on reboxetine’s steady state pharmacological properties^7^. However, the variation on binding modes of 2 enantiomers with hNET remains elusive. In this work, a collective computational method was applied to identify binding modes of 2 enantiomers and distinguish their conformational variations in hNET. As shown in SI, Fig. S13, interactions of salt bridge, hydrogen bond and hydrophobic contact between sNRIs and 11 hot spot residues were essential for both enantiomers in hNET’s recognition, which could be further analyzed by the energy decomposition of those 2 enantiomers (SI, Fig. S14).

**Figure 7:**
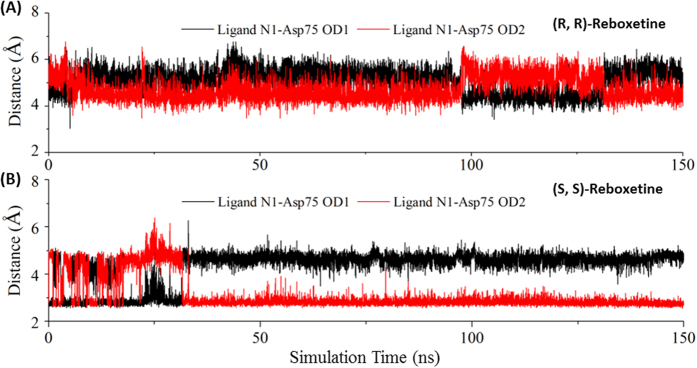
Distance between the ligand N1 and Asp75 OD in (**A**) (R, R)-reboxetine and (**B**) (S, S)-reboxetine binding to hNET during the entire 150 ns MD simulation.

**Figure 8:**
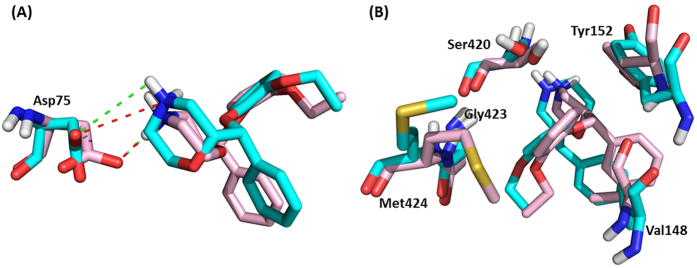
Superimposition of and conformation variation between (R, R)-reboxetine (in cyan) and (S, S)-reboxetine (in light pink) together with their interacting residues (in corresponding color). (**A**) Conformational changes of ligands and residue Asp75 were displayed; (**B**) conformational changes of ligands and residues Val148, Tyr152, Ser420, Gly423 and Met424 were illustrated. The salt bridge interaction was displayed in red dashed line and the hydrogen bond interaction was in green.

However, the calculated binding free energies of (R, R)-reboxetine and (S, S)-reboxetine were −34.69 and −47.67 kcal/mol, respectively, which were consistent with the experimental results^7^. To understand the difference in binding affinities of 2 enantiomers, per-residue energy contribution analysis was performed to find out residues with significant variation in energy contribution (absolute variation ≥0.5 kcal/mol) between 2 enantiomers. In this work, 6 residues (Asp75, Val148, Tyr152, Ser420, Gly423 and Met424) favoring the binding of (S, S)-reboxetine over (R, R)-reboxetine were identified. For the residue Asp75, the average interaction distance between the polar nitrogen of (S, S)-reboxetine and the carboxyloxygen of Asp75 was 2.8 Å, while the distance was 4.2 Å for (R, R)-reboxetine (Fig. 7). A shorter interaction distance for (S, S)-reboxetine guaranteed a much stronger salt bridge and hydrogen bond interaction than (R, R)-reboxetine. Meanwhile (S, S)-reboxetine formed strong hydrophobic interactions with other 5 residues (Val148, Tyr152, Ser420, Gly423 and Met424). Comparing to (S, S)-reboxetine’s binding, (R, R)-reboxetine and those 5 residues suffered from obvious conformation shifts (Fig. 8), which significantly increased the hydrophobic interaction distance between the ligand and residues.

## Conclusion

In this study, multiple computational methods were integrated to explore the inhibitory mechanism of approved sNRIs (atomoxetine, maprotiline, reboxetine and viloxazine). First, a recently reported co-crystal structure of *drosophila* dopamine transporter (dDAT) in complex with reboxetine was utilized to construct the homology model of hNET. Then, those studied sNRIs were docked into hNET for MD simulation. 3 lines of evidences were further provided to verify the simulation results. Consequently, a binding mode shared by approved sNRIs was discovered by clustering the binding free energies of residues. Eleven residues (Phe72, Asp75, Ala145, Val148, Gly149, Tyr152, Phe317, Phe323, Ser419, Ser420, Gly423) in hNET were identified as crucial for revealing the binding mode of sNRIs-hNET complex. Moreover, the binding modes of reboxetine’s enantiomers with hNET were compared, and 6 residues (Asp75, Val148, Tyr152, Ser420, Gly423 and Met424) favoring the binding of (S, S)-reboxetine over that of (R, R)-reboxetine were identified. This is the first study reporting that those 11 residues are the common determinants for the binding of approved sNRIs. The identified binding mode shed light on the binding mechanism of approved sNRIs, and might therefore help identify novel scaffolds with improved drug efficacy.

## Additional Information

**How to cite this article**: Zheng, G. *et al*. Exploring the Inhibitory Mechanism of Approved Selective Norepinephrine Reuptake Inhibitors and Reboxetine Enantiomers by Molecular Dynamics Study. *Sci. Rep.* **6**, 26883; doi: 10.1038/srep26883 (2016).

## Supplementary Material

Supplementary Information

## Figures and Tables

**Figure 1:**
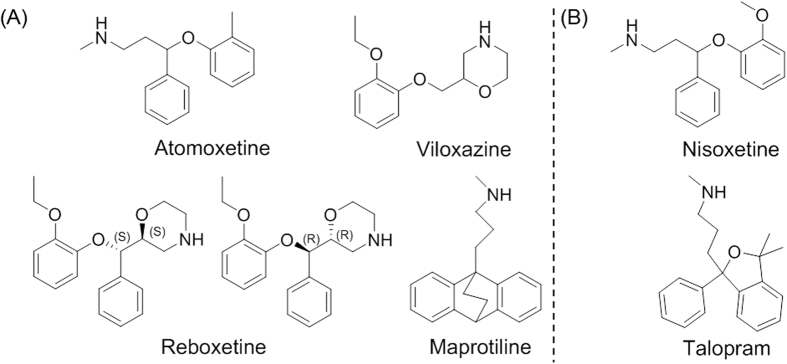
Structures of 6 sNRIs studied in this work. (**A**) 4 currently marketed sNRIs approved by either the U. S. FDA (atomoxetine and reboxetine) or the European Medicines Agency (maprotiline and viloxazine); (**B**) 2 standard sNRIs (nisoxetine and talopram) widely used in scientific research.

**Table 1:** Comparison of binding free energies between the calculated results and the experimental data of 6 sNRIs of this study binding to hNET.

sNRIs studied	K_i_^a^	ΔG_exp_^b^	ΔΔG_exp_^c^	ΔG_MM/GBSA_^d^	ΔΔG_calc_^c^
Atomoxetine	5.00	−11.33	−1.60	−41.42 ± 0.13	−3.94
Maprotiline	7.00	−11.13	−1.39	−40.21 ± 0.09	−2.73
Nisoxetine	1.60	−12.00	−2.26	−46.05 ± 0.13	−8.57
Talopram	2.90	−11.65	−1.91	−42.47 ± 0.12	−4.99
(S, S)-reboxetine	0.08	−13.78	−4.04	−47.67 ± 0.12	−10.19
Viloxazine	73.00	−9.74	0.00	−37.48 ± 0.12	0.00

ΔG is in kcal/mol and K_i_ value is in nM.

^a^The medium experimental K_i_ values^7^,^27^,^69^,^70^,^71^.

^b^Estimated binding free energy based on K_i_ values by the equation ΔG_exp_ = RTln (K_i_).

^c^ΔΔ**G** is defined as the change of binding free energy (ΔG) using viloxazine as a reference.

^d^Calculated MM/GBSA binding free energies with the standard error of the mean (the standard deviation divided by the square root of the number of snapshots) in this work.

**Table 2:** Comparison of binding free energies between the calculated results and the experimental data of 6 sNRIs-hNET complexes before and after hDAT-like mutations in hNET^76^ (ΔG is in kcal/mol).

sNRIs studied	hDAT-like mutations in the S1 site^76^	Calculated values		Experimental values^76^	
		ΔΔG_calc_^a^	Fold-change of potency^b^	Fold-change of potency^c^	ΔΔG_exp_^d^
Talopram	S145S-Y151F-I315V-F316C-S420A-A426S	3.70	514.09	207.00 (151.00~284.00)	3.16 (2.98~3.35)
Nisoxetine		2.89	131.08	168.42 (107.73~251.88)	3.04 (2.78~3.28)
Atomoxetine		1.15	6.96	27.87 (18.47~40.15)	1.97 (1.73~2.19)
Reboxetine		0.47	2.70	10.27 (7.18~14.31)	1.38 (1.17~1.58)
Maprotiline		2.45^e^	62.39^e^	–e	–e
Viloxazine		1.09^e^	6.29^e^	–e	–e

Detail information of each energy term can be found in SI, Table S3.

^a^ΔΔG_calc_ = ΔG_mutation_ − ΔG_wild type_.

^b^Fold-changes of potency were derived from ΔΔG_calc_ by the equation ΔΔG_calc_ = RTln (FC_potency_).

^c^Fold-changes of potency measured by K_i_ values (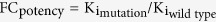)^76^. Numbers out of the bracket indicated the fold-changes derived from the medium experimental values of both 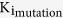 and 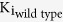. The first number in the bracket indicated the minimum fold-changes, while the second one indicated the maximum fold-changes.

^d^ΔΔG_exp_ were derived from the FC_potency_ by the equation ΔΔG_exp_ = RTln (FC_potency_).

^e^Not included in Andersen’s experimental study, but simulated in this work^76^.

**Table 3:** The calculated and experimental changes in binding free energies of 8 sNRIs-hNET complexes (2 sNRIs against 4 single-point mutations) before and after those mutations in hNET^25^ (ΔG is in kcal/mol).

sNRIs studied	Single point mutations in hNET	Calculated value		Experimental value^25^	
		ΔΔG_calc_^a^	Fold-change of potency^b^	Fold-change of potency^c^	ΔΔG_exp_^d^
Atomoxetine	F72Y	−0.03	0.95	0.78 (0.55~1.14)	−0.15 (−0.35~0.08)
	N153S	−0.31	0.59	2.33 (1.73~3.29)	0.50 (0.33~0.71)
	F323Y	1.93	25.95	3.89 (2.45~6.14)	0.81 (0.53~1.08)
	S419T	1.27	8.52	12.67 (8.73~18.86)	1.51 (1.28~1.74)
Maprotiline	F72Y	−0.31	0.59	0.59 (0.34~0.99)	−0.31 (−0.64~−0.01)
	N153S	0.05	1.09	2.34 (1.44~3.75)	0.50 (0.22~0.78)
	F323Y	0.96	5.05	4.97 (3.21~7,75)	0.95 (0.69~1.21)
	S419T	1.36	9.92	5.88 (3.61~9.43)	1.05 (0.76~1.33)

Detail information of each energy term can be found in SI, Table S4.

^a^ΔΔG_calc_ = G_mutation_ − ΔG_wild type_.

^b^Fold-changes of potency were derived from ΔΔG_calc_ by the equation ΔΔG_calc_ = RTln (FC_potency_).

^c^Fold-changes of potency measured by K_i_ values 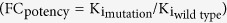
25. Numbers out of the bracket indicated the fold-changes derived from the medium experimental values of both 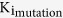 and 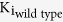. The first number in the bracket indicated the minimum fold-changes, while the second one indicated the maximum fold-changes.

^d^ΔΔG_exp_ were derived from the FC_potency_ by the equation ΔΔG_exp_ = RTln (FC_pontency_).
