# Supplementary material for: Exploring the Inhibitory Mechanism of Approved Selective Norepinephrine Reuptake Inhibitors and Reboxetine Enantiomers by Molecular Dynamics Study
Source: Sci Rep. 2016 May 27;6:26883. doi: 10.1038/srep26883 (PMC4882549; doi:10.1038/srep26883)
Supplement: Supplementary Information [file srep26883-s1.pdf]

Supporting Information for:

Exploring the Inhibitory Mechanism of Approved Selective Norepinephrine Reuptake Inhibitors and Reboxetine Enantiomers by Molecular Dynamics Study

Guoxun Zheng[1], Weiwei Xue[1,*], Panpan Wang[1], Fengyuan Yang[1], Bo Li[1], Xiaofeng Li[1], Yinghong Li[1], Xiaojun Yao[2], Feng Zhu[1,*]

[1] Innovative Drug Research and Bioinformatics Group, Innovative Drug Research Centre and School of Pharmaceutical Sciences, Chongqing University, Chongqing 401331, China

[2] State Key Laboratory of Applied Organic Chemistry and Department of Chemistry, Lanzhou University, Lanzhou 730000, China

* Corresponding authors: Feng Zhu (zhufeng@cqu.edu.cn) and Weiwei Xue (xueww@cqu.edu.cn)

Supporting Information for METHODS

Molecular Docking

To get the initial binding mode of 6 studied sNRIs (atomoxetine, maprotiline, (S, S)-reboxetine, viloxazine, nisoxetine and talopram) to hNET, the molecular docking was carried out by the *Glide software*[1] with default settings of standard precision mode. The 3D structures of these 6 sNRIs were retrieved from the PubChem database. Then, their structures were preprocessed by *LigPrep*[2] using OPLS-2005 force field[3] and resulted in a low energy conformation. The ionized state was assigned by *Epik*[4] at a pH value of 7.0 ±2.0.

To prepare hNET structure for docking, reboxetine from the dDAT co-crystal structure (PDB entry 4XNX[5]) was introduced to the hNET model by *PyMOL*'s[6] structure superimposition. Then, the *Protein Preparation Wizard* module in *Maestro*[7] was used to add hydrogen atoms, assign partial charges using OPLS-2005 force field[3], assign protonation states and minimize the structure. The minimization was terminated when the root mean square deviation reached the maximum value of 0.30Å. The docking grid box was defined by centering on reboxetine in the hNET model using the *Receptor Grid Generation* tool in the *Glide*. In molecular docking, the protein was fixed while the ligands were flexible. In detail, 5000 poses were generated during the initial phase of the docking calculation, out of which best 400 poses were chosen for energy minimization by 100 steps of conjugate gradient minimizations.

Cross Docking

To guarantee the docking effective, the X-ray structures of dDAT in complex with reboxetine and nisoxetine (PDB code 4XNX[5] and 4XNU[5] were used for cross docking by the *Glide software*[1]. The ligands and protreins were firstly prepared in the same way as described above. Then, the docking grid boxes were defined by centering on reboxetine or nisoxetine in dDAT using the *Receptor Grid Generation* tool in the *Glide*[1]. Finally, the prepared reboxetine and nisoxetine were cross docked into the corresponding nisoxetine and reboxetine bound dDAT structures using the same parameter settings for 6 studied sNRIs' molecular docking.

Protein-Ligand/Membrane System Setup

Coordinates of 6 obtained sNRIs-hNET structures were pre-oriented in *OPM*[8] with respect to the Membrane Normal which is defined by the Z-axis. Then NRIs-hNET complexes were embedded into the explicit POPC lipid bilayer using the *Membrane Builder module* of *CHARMM-GUI*[9].

The TIP3P water[10] of 20Å thickness was placed above and below the membrane and the salt

concentration was kept at 0.15M by adding Na^+ and Cl^-. The overall system contained a total of ~96,400 atoms per periodic cell. The box size was set as $83Å \times 83Å \times 127Å$.

MD simulation

MD simulation was performed within the *AMBER14*[11] using GPU-accelerated *PMEMD* on 16 cores of an array of two 2.6GHz Intel Xeon E5-2650v2 processors and 4 pieces of NVDIA Tesla NVIDIA Tesla K20C graphics card.

AMBER force field *ff14SB*[12] and *Lipid14*[13] were used for protein and lipids, respectively. The ions parameters for TIP3P water were collected from Joung & Cheatham[14]. The force field parameters for atomoxetine, maprotiline, reboxetine, viloxazine, nisoxetine, talopram and cholesterol were described by the *General AMBER Force Field* (GAFF)[15] and the charges were assigned using the *Restrained Electrostatic Potential partial charges*[16] with *Antechamber*[17]. Geometry optimization and electrostatic potential calculations were performed with *Gaussian09* at the HF/6-31G* level[18] .

Prior to MD simulation, the prepared systems were subjected to initial energy minimization by two steps. The first step is to apply harmonic restraints with a force constant of 10.0 kcal/(mol ·$Å^2$) to the lipid and solute atoms, and the second step is to allow all atoms to move freely. In each step, energy minimization was performed by the steepest descent method for the first 5,000 steps and the conjugated gradient method for the subsequent 5,000 steps. After the initial minimization, the system was heated through two sequential runs to 310K while keeping the lipid and solute atoms fixed over 100ps in the NVT ensemble. Firstly, the system is heated to 100K and then gradually to 310K. Subsequently, 10 times unconstrained equilibration (5ns) at 310K were performed to equilibrate the system's periodic boundary condition. Finally, 150ns MD simulation was conducted in NPT ensemble under a temperature of 310K and a pressure of 1 atm. Temperature is controlled here using *Langevin dynamics* while pressure is controlled using *anisotropic Monte Carlo barostat* included in *AMBER14*[11].

In the MD simulations, periodic boundary conditions were employed and direct space interaction was calculated by considering the long range electrostatic interaction (cutoff = 10.0Å) using *particle-mesh Ewald* method[19]. Here, the dimension of periodic box was measured using *VMD 1.9.1*[20]. All bonds involving hydrogen atoms were constrained with the *SHAKE algorithm*[21] allowing an integration time step of 2fs.

All MD trajectories analysis, such as the root mean square deviation between structure pairs and the extraction of representative structures from trajectories, were performed using *cpptraj*[22] as

implemented in *AMBER14*[11]. Visualization of structures was performed with *PyMOL*[6].

Binding Free Energy Calculation

The relative binding free energies of sNRIs on the hNET were calculated using the single-trajectory based method of *molecular mechanics/generalized Born surface area* (MM/GBSA)[23, 24]. The *mm_pbsa.pl* under *AMBER14*[11] was used to carry out the MM/GBSA calculation. A total of 500 snapshots were taken from the last 50ns equilibrium trajectory of each complex. For each snapshot, all ions (except for the functional ions in the binding site), lipid, and water molecules were removed. The MM/GBSA binding free energy calculated by excluding entropic contribution is given by

$$\Delta \mathrm{G}_{\mathrm{MM/GBSA}} = \Delta \mathrm{E}_{\mathrm{vdW}} + \Delta \mathrm{E}_{\mathrm{ele}} + \Delta \mathrm{G}_{\mathrm{pol}} + \Delta \mathrm{G}_{\mathrm{nonpol}} \quad (1)$$

In Eq. (1), $\Delta \mathrm{E}_{\mathrm{vdW}}$ and $\Delta \mathrm{E}_{\mathrm{ele}}$ represent the van der Waals and electrostatic components in gas phase, and $\Delta \mathrm{G}_{\mathrm{pol}}$ and $\Delta \mathrm{G}_{\mathrm{nonpol}}$ stand for polar and non-polar solvent interaction energies. $\Delta \mathrm{E}_{\mathrm{vdW}}$ and $\Delta \mathrm{E}_{\mathrm{ele}}$ were calculated using the *AMBER* force field *ff14SB*[12], and the electrostatic free energy of solvation ($\Delta \mathrm{G}_{\mathrm{pol}}$) were calculated by the modified GB model (igb = 2) developed by Onufriev et al[25]. The solute and solvent dielectric constants were set to 2 and 80, respectively. The nonpolar solvation free energy ($\Delta \mathrm{G}_{\mathrm{nonpol}}$) was calculated from the solvent accessible area (SASA) using the method of *linear combination of pairwise overlaps* (LCPO) ($\Delta \mathrm{G}_{\mathrm{nonpol}} = 0.0072 \times \Delta SASA$)[26]. The SASA here was determined with probe radii of 1.4Å.

Per-residue Free Energy Decomposition Analysis

To quantitatively evaluate the contribution to sNRIs' binding, the total binding free energy was decomposed on a per-residue basis, which including contributions from the van der Waals term ($\Delta \mathrm{E}_{\mathrm{vdW}}^{\mathrm{per-residue}}$), the electrostatic term ($\Delta \mathrm{E}_{\mathrm{ele}}^{\mathrm{per-residue}}$), the polar term ($\Delta \mathrm{G}_{\mathrm{pol}}^{\mathrm{per-residue}}$) and the nonpolar term ($\Delta \mathrm{G}_{\mathrm{nonpol}}^{\mathrm{per-residue}}$) for the ligand and each residue, as shown in Eq. (2):

$$\Delta \mathrm{G}_{\mathrm{MM/GBSA}}^{\mathrm{per-residue}} = \Delta \mathrm{E}_{\mathrm{vdW}}^{\mathrm{per-residue}} + \Delta \mathrm{E}_{\mathrm{ele}}^{\mathrm{per-residue}} + \Delta \mathrm{G}_{\mathrm{pol}}^{\mathrm{per-residue}} + \Delta \mathrm{G}_{\mathrm{nonpol}}^{\mathrm{per-residue}} \quad (2)$$

where $\Delta \mathrm{E}_{\mathrm{vdW}}^{\mathrm{per-residue}}$, $\Delta \mathrm{E}_{\mathrm{ele}}^{\mathrm{per-residue}}$ and $\Delta \mathrm{G}_{\mathrm{pol}}^{\mathrm{per-residue}}$ were calculate using the same approach in the *Binding Free Energy Calculation* section of the paper, while the non-polar term was estimated as $\Delta \mathrm{G}_{\mathrm{nonpol}}^{\mathrm{per-residue}} = 0.0072 \times \Delta SASA$ based on the recursive approximation of a sphere around an atom, starting from an icosahedron (ICOSA)[11].

Supporting Information for RESULTS AND DISCUSSION

Analysis of Salt Bridge and Hydrogen Bond Interaction

Further analysis on energy contributions of sNRIs' different chemical groups reveals a vital role of chemical group R_1 in sNRIs and hNET recognition[27]. R_1 interacts with hNET by salt bridge to Asp75 and hydrogen bond to Phe72 and Phe317, which in total consists of 34.29%, 23.04%, 28.04% and 22.70% of binding free energies for atomoxetine, maprotiline, (S, S)-reboxetine and viloxazine, respectively. To further understand these interactions anchoring different sNRIs into the binding site, salt bridge and hydrogen bond were monitored along the entire MD simulation.

Salt bridge formed by the positively charged nitrogen of sNRIs and the carboxyl group of Asp75 was vital to the sNRIs-hNET's recognition. As illustrated in **SI**, **Figure S14A**, **S14B** and **S14C**, the salt bridge (N^+···OD Asp75) in atomoxetine, maprotiline and (S, S)-reboxetine's binding was relatively stable, while the salt bridge in viloxazine's binding fluctuates significantly during the simulation (**SI**, **Figure S14D**).

Hydrogen bond formed between 4 approved sNRIs and Phe72, Asp75 and Phe317 were also monitored along the entire MD simulation. As shown in **Table S2**, hydrogen bond between Asp75 and amine hydrogen of all 4 approved sNRIs was formed. Occupancy values further demonstrated a stable hydrogen bond along the simulation with interaction distance of ~2.80Å and bond angle ranging from 155 °to 160 °. Hydrogen bond between Phe72 and amine hydrogen was formed in atomoxetine, (S, S)-reboxetine and viloxazine, while hydrogen bond between Phe317 and amine hydrogen could only be found in maprotiline and viloxazine.

Supporting Information for TABLES

Table S1. The calculated and experimental binding free energies of 6 studied sNRIs binding to hNET (ΔG is in kcal/mol and K_i value is in nM)

sNRIs	Calculated value						Experimental value		
	ΔE_{ele}	ΔE_{vdw}	ΔG_{pol}	ΔG_{nonpol} [a]	ΔG_{MMGBSA} [b]	$\Delta\Delta \mathbf{G}_{calc}$ [c]	ΔG_{exp}	$\Delta\Delta \mathbf{G}_{exp}$	K_i
Atomoxetine	-40.75 ±0.19	-39.13 ±0.11	43.94 ±0.18	-5.47 ±0.01	-41.42 ±0.13	-3.94	-11.33	-1.60	5.00[d]
Maprotiline	-26.94 ±0.19	-42.03 ±0.11	33.85 ±0.16	-5.09 ±0.00	-40.21 ±0.09	-2.73	-11.13	-1.39	7.00[e]
Nisoxetine	-56.87 ±0.18	-41.56 ±0.13	52.37 ±0.14	-5.85 ±0.01	-46.05 ±0.13	-8.57	-12.00	-2.26	1.60[f]
Talopram	-24.47 ±0.19	-43.43 ±0.11	25.43 ±0.15	-5.49 ±0.01	-42.47 ±0.12	-4.99	-11.65	-1.91	2.90[g]
Reboxetine	-34.13 ±0.19	-45.67 ±0.13	38.44 ±0.18	-6.30 ±0.01	-47.67 ±0.12	-10.19	-13.78	-4.03	0.08[h]
Viloxazine	-31.88 ±0.40	-36.08 ±0.11	35.13 ±0.36	-4.65 ±0.01	-37.48 ±0.12	0.00	-9.74	0.00	73.00[e]

[a] Estimated binding free energy based on experimental K_i values by $\Delta G_{exp} = RT\ln(K_i)$.

[b] Calculated MM/GBSA binding free energies with the standard error of the mean (the standard deviation divided by the square root of the number of snapshots) in this work.

[c] $\Delta\Delta G$ is defined as the change of binding free energy (ΔG) using the viloxazine as a reference.

[d-h] The medium experimental K_i values[28-32].

Table S2. Hydrogen bond network analysis

Drugs	Acceptor	Donor	Distance (Å) [a]	Angle (°) [a]	Occupancy (%) [b]
Atomoxetine	PHE72 (O)	Atomoxetine (N1-H22)	2.80	154.50	62.40
	ASP75 (OD1)	Atomoxetine (N1-H3)	2.78	155.30	29.58
	ASP75 (OD2)	Atomoxetine (N1-H3)	2.78	154.83	25.71
Maprotiline	ASP75 (OD2)	Maprotiline (N1-H23)	2.82	158.01	79.11
	ASP75 (OD1)	Maprotiline (N1-H23)	2.82	155.27	5.71
	PHE317 (O)	Maprotiline (N1-H24)	2.83	149.60	14.21
Reboxetine	PHE72 (O)	Reboxetine (N1-H23)	2.80	156.19	77.97
	ASP75 (OD2)	Reboxetine (N1-H24)	2.78	160.08	77.33
	ASP75 (OD1)	Reboxetine (N1-H24)	2.78	158.55	14.09
Viloxazine	ASP75 (OD1)	Viloxazine (N1-H20)	2.80	159.46	57.12
	PHE317 (O)	Viloxazine (N1-H19)	2.80	149.80	36.76
	PHE72 (O)	Viloxazine (N1-H19)	2.78	143.85	11.21

[a] The hydrogen bonds listed above are those with acceptor ··· donor atom distance less than 3.5Å and acceptor ··· H-donor angle greater than 120°.

[b] Occupancy (%): to evaluate the stability and the strength of the hydrogen bond.

Table S3. The calculated and experimental changes in binding free energies of 6 sNRIs-hNET complexes before and after hDAT-like mutations (S145S-Y151F-I315V-F316C-S420A-A426S) in hNET[33] (ΔG is in kcal/mol)

sNRIs studied	Calculated value						Experimental value	
	ΔE_{ele}	ΔE_{vdw}	ΔG_{pol}	ΔG_{nonpol}	ΔG_{MMGBSA}	$\Delta\Delta \mathbf{G}_{cal}$ [a]	Fold-change of potency [b]	$\Delta\Delta \mathbf{G}_{exp}$ [c]
Talopram	-30.59 ±0.16	-41.23 ±0.14	38.44 ±0.14	-5.39 ±0.01	-38.77 ±0.16	3.70	207.00 (151.00~284.00)	3.16 (2.98~3.35)
Nisoxetine	-43.77 ±0.19	-41.00 ±0.13	41.61 ±0.17	-6.00 ±0.01	-43.16 ±0.12	2.89	168.42 (107.73~251.88)	3.04 (2.78~3.28)
Atomoxetine	-33.25 ±0.13	-37.79 ±0.16	33.20 ±0.10	-5.42 ±0.01	-40.27 ±0.10	1.15	27.87 (18.47~40.15)	1.97 (1.73~2.19)
Reboxetine	-38.15 ±0.17	-44.70 ±0.12	35.65 ±0.15	-5.63 ±0.01	-47.20 ±0.12	0.47	10.27 (7.18~14.31)	1.38 (1.17~1.58)
Maprotiline	-33.52 ±0.16	-37.24 ±0.11	33.00 ±0.13	-5.14 ±0.01	-37.76 ±0.10	2.45 [d]	-- [d]	-- [d]
Viloxazine	-36.45 ±0.14	-33.64 ±0.12	33.71 ±0.13	-4.85 ±0.01	-36.39 ±0.10	1.09 [d]	-- [d]	-- [d]

[a] $\Delta\Delta G_{cal} = \Delta G_{mutation} - \Delta G_{wild\ type}$ (the $\Delta G_{wild\ type}$ of each studied sNRI was listed in **Table 1** and **SI, Table S1**).

[b] Fold-changes of potency measured by K_i values ($FC_{potency} = {K_i}_{mutation}/{K_i}_{wild\ type}$)[33]. Numbers out of the bracket indicated the fold-changes derived from the medium experimental values of both ${K_i}_{mutation}$ and ${K_i}_{wild\ type}$. The first number in the bracket indicated the minimum fold-changes, while the second one indicated the maximum fold-changes.

[c] $\Delta\Delta G_{exp}$ were derived from the $FC_{potency}$ by the equation $\Delta\Delta G_{exp} = RTln(FC_{potency})$.

[d] Not included in Andersen's experimental study, but simulated in this work[33].

Table S4. The calculated and experimental changes in binding free energies of 8 sNRIs-hNET complexes (2 sNRIs against 4 single-point mutations) before and after those mutations in hNET[27] (ΔG is in kcal/mol)

sNRIs studied	Single point mutation	Calculated value						Experimental value	
		ΔE_{ele}	ΔE_{vdw}	ΔG_{pol}	ΔG_{nonpol}	ΔG_{MMGBSA}	$\Delta\Delta \boldsymbol{G}_{\boldsymbol{cal}}$ [a]	Fold-change of potency [c]	$\Delta\Delta \boldsymbol{G}_{\boldsymbol{exp}}$ [b]
Atomoxetine	F72Y	-39.92 $\pm$0.11	-39.43 $\pm$0.17	37.90 $\pm$0.10	-5.39 $\pm$0.01	-41.45 $\pm$0.10	-0.03	0.78 (0.55~1.14)	-0.15 (-0.35~0.08)
	N153S	-40.70 $\pm$0.13	-39.21 $\pm$0.11	38.20 $\pm$0.10	-5.50 $\pm$0.01	-41.71 $\pm$0.10	-0.31	2.33 (1.73~3.29)	0.50 (0.33~0.71)
	F323Y	-37.86 $\pm$0.09	-37.69 $\pm$0.07	36.08 $\pm$0.06	-5.06 $\pm$0.01	-39.47 $\pm$0.07	1.93	3.89 (2.45~6.14)	0.81 (0.53~1.08)
	S419T	-38.16 $\pm$0.09	-39.15 $\pm$0.07	37.18 $\pm$0.06	-5.26 $\pm$0.01	-40.13 $\pm$0.10	1.27	12.67 (8.73~18.86)	1.51 (1.28~1.74)
Maprotiline	F72Y	-29.64 $\pm$0.20	-41.89 $\pm$0.12	35.22 $\pm$0.16	-5.23 $\pm$0.01	-40.52 $\pm$0.11	-0.31	0.59 (0.34~0.99)	-0.31 (-0.64~-0.01)
	N153S	-24.96 $\pm$0.17	-41.67 $\pm$0.11	25.48 $\pm$0.14	-5.06 $\pm$0.01	-40.16 $\pm$0.10	0.05	2.34 (1.44~3.75)	0.50 (0.22~0.78)
	F323Y	-26.99 $\pm$0.10	-39.27 $\pm$0.07	27.00 $\pm$0.05	-4.70 $\pm$0.01	-39.25 $\pm$0.10	0.96	4.97 (3.21~7,75)	0.95 (0.69~1.21)
	S419T	-23.93 $\pm$0.18	-40.81 $\pm$0.11	24.89 $\pm$0.14	-4.99 $\pm$0.01	-38.85 $\pm$0.10	1.36	5.88 (3.61~9.43)	1.05 (0.76~1.33)

[a] $\Delta\Delta G_{cal} = \Delta G_{mutation} - \Delta G_{wild\ type}$ (the $\Delta G_{wild\ type}$ of each studied sNRI was listed in **Table 1** and **SI, Table S1**).

[b] Fold-changes of potency measured by K_i values ($FC_{potency} = K_{i_{mutation}}/K_i$)[27]. Numbers out of the bracket indicated the fold-changes derived from the medium experimental values of both $K_{i_{mutation}}$ and $K_{i_{wild\ type}}$. The first number in the bracket indicated the minimum fold-changes, while the second one indicated the maximum fold-changes.

[c] $\Delta\Delta G_{exp}$ were derived from the $FC_{potency}$ by the equation $\Delta\Delta G_{exp} = RT\ln(FC_{potency})$.

Supporting Information for FIGURES

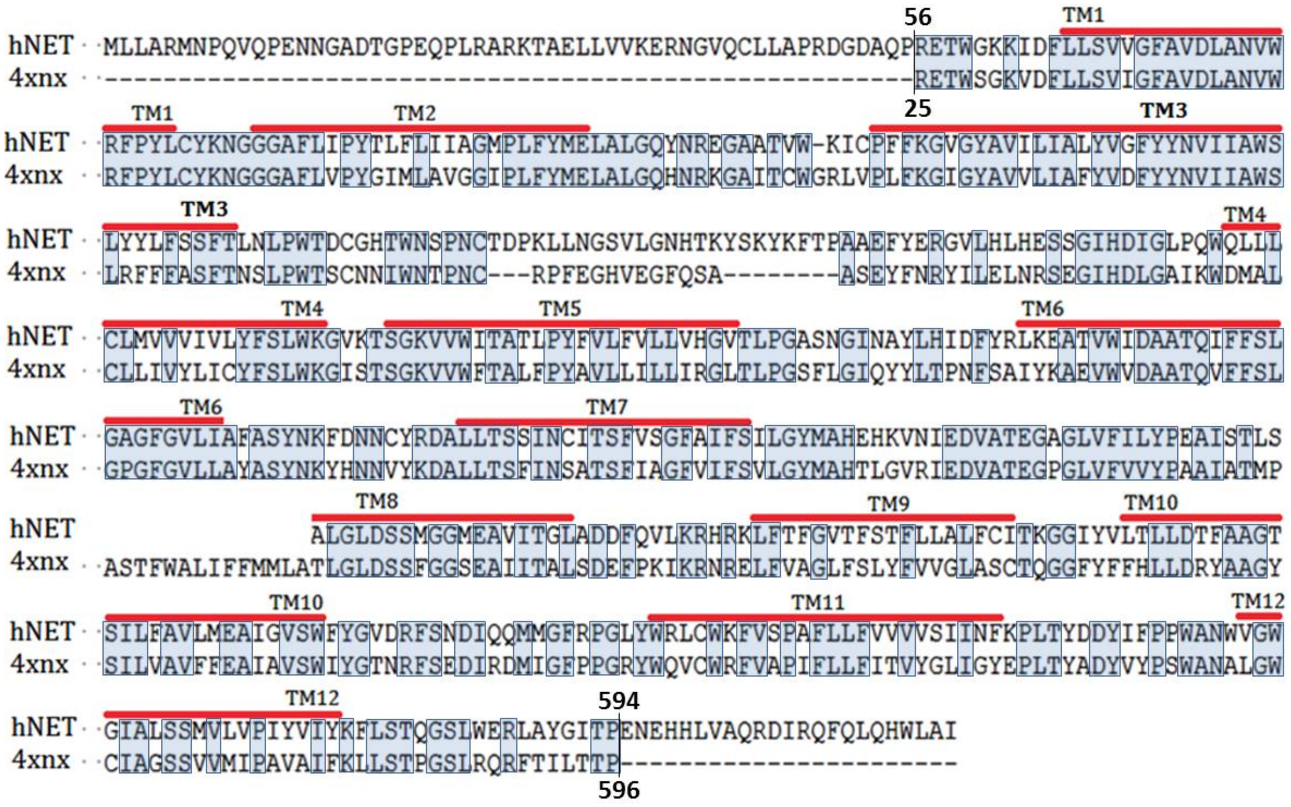

Figure S1. Sequence alignment between hNET (from Arg56 to Pro594) and dDAT (from Arg25 to Pro596). The 12 transmembrane (TM1 to TM12) alpha helices were labeled above the sequence. The sequence of hNET was collected from the UniProt database (Accession number: P23975).

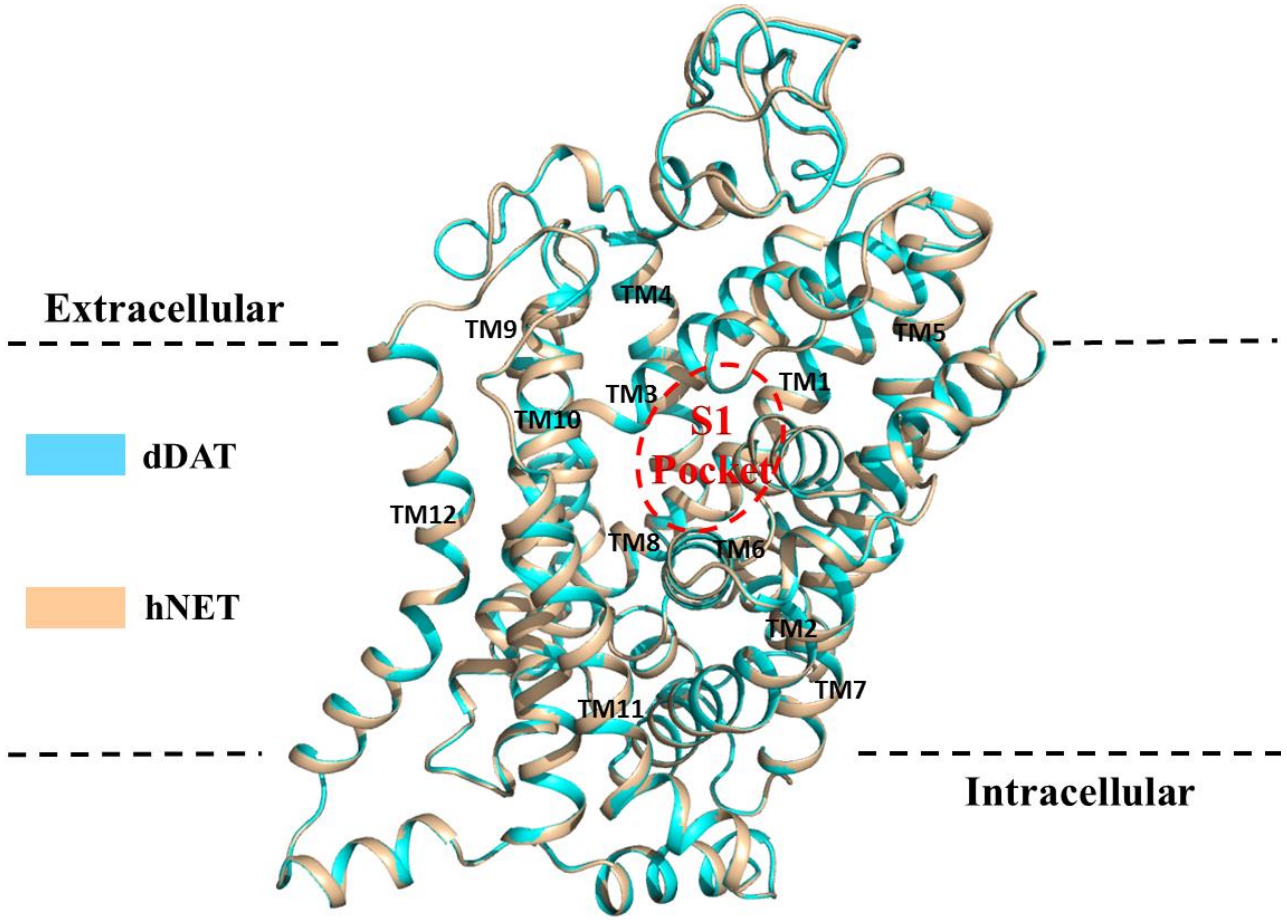

Figure S2. Structural superimposition between the homology model of hNET and the crystal structure of dDAT (PDB entry 4XNX[5]). The hNET and dDAT were displayed in wheat and cyan.

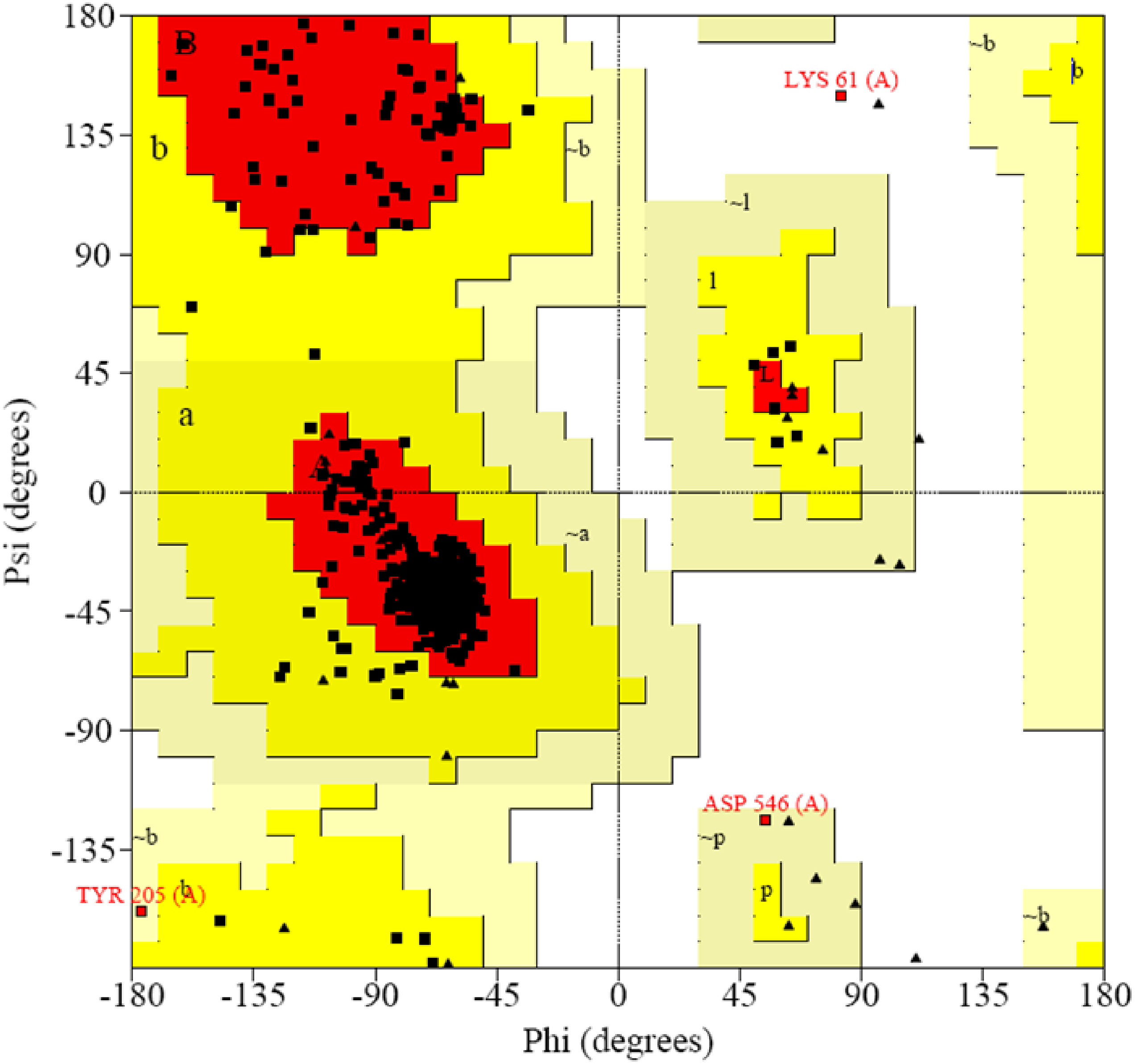

Plot statistics

Residues in most favoured regions [A,B,L]	443	93.7%
Residues in additional allowed regions [a,b,l,p]	27	5.7%
Residues in generously allowed regions [~a,~b,~l,~p]	2	0.4%
Residues in disallowed regions	1	0.2%
	----	------
Number of non-glycine and non-proline residues	473	100.0%
Number of end-residues (excl. Gly and Pro)	1	
Number of glycine residues (shown as triangles)	46	
Number of proline residues	19	

Total number of residues	539	

Figure S3. Ramachandran plot of the hNET model.

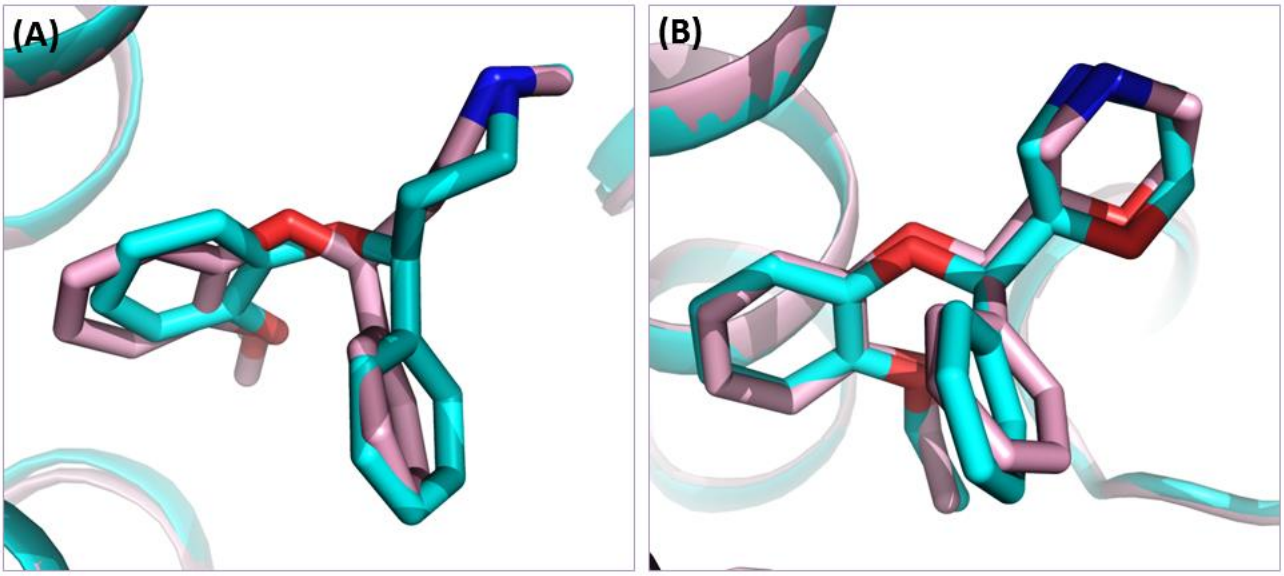

Figure S4. Structural superimposition between the cross-docking pose (light pink) of (A) nisoxetine into the structure of reboxetine bound dDAT and (B) reboxetine into the structure of reboxetine bound dDAT with their corresponding co-crystal poses (cyan) in dDAT.

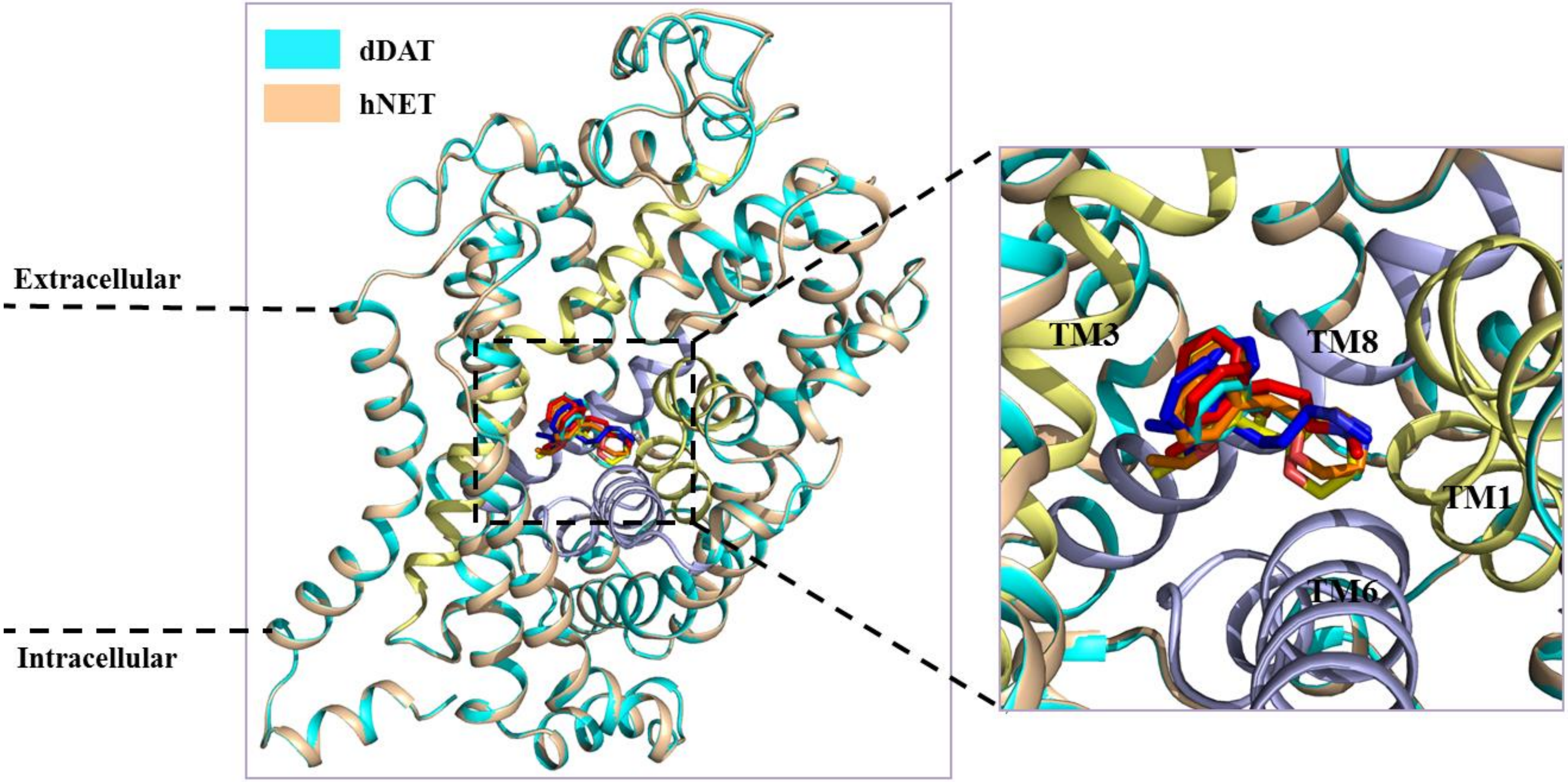

Figure S5. Superimposition of the docked poses of 6 studied sNRIs atomoxetine (cyan), maprotiline (light pink), nisoxetine (red), talopram (blue), reboxetine (orange) and viloxazine (green) in the binding site of modeled hNET with the pose of the co-crystallized reboxetine (yellow) in dDAT (PDB entry 4XNX[5]). The binding site is primarily surrounded by TM1, TM3, TM6 and TM8. TM1 and TM3 were highlighted in pale yellow and TM6 and TM8 are highlighted in light blue. The 6 sNRIs accommodate the binding site with a similar orientation to the pose of the co-crystallized reboxetine.

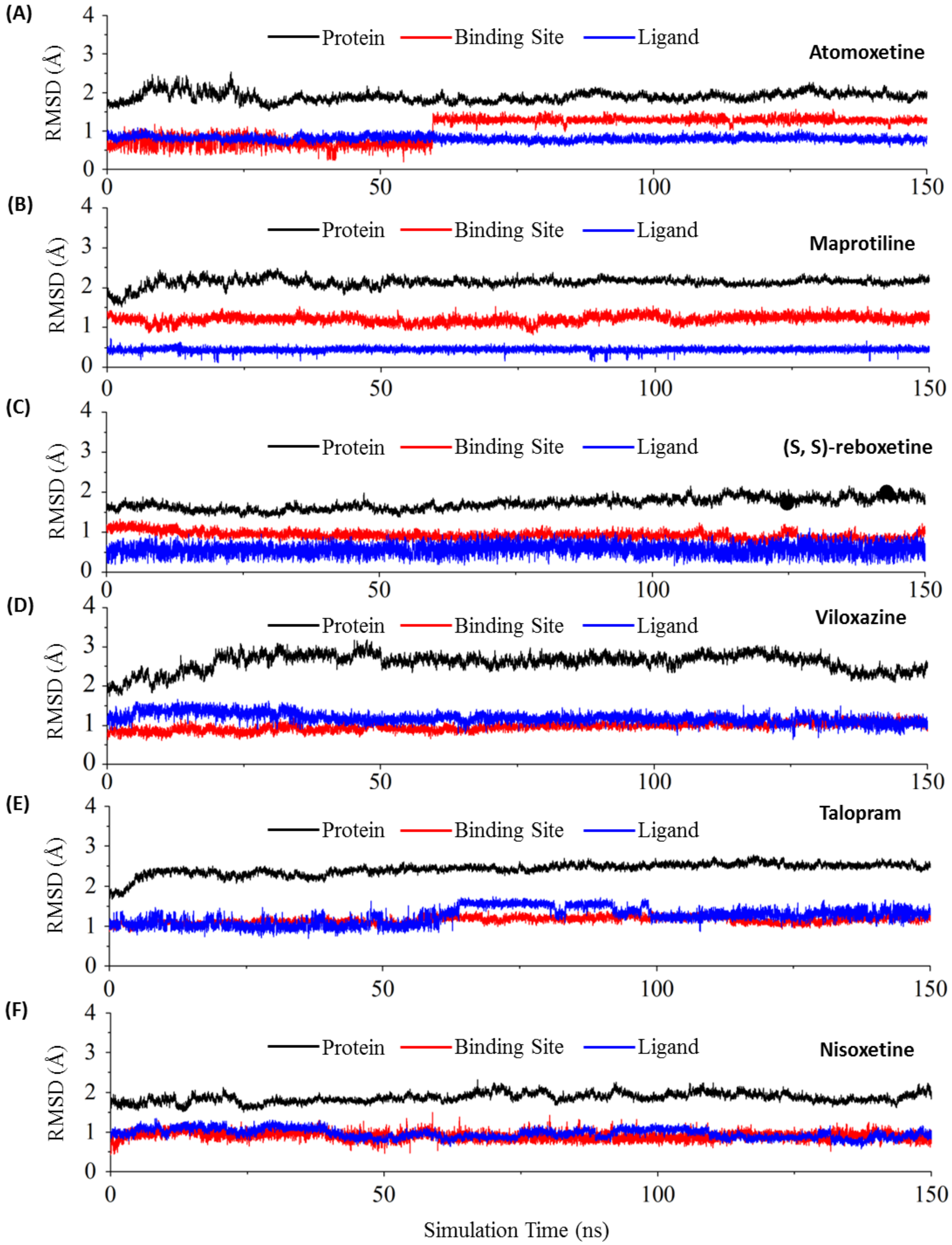

Figure S6. RMSDs of protein backbone atoms (black), ligand heavy atoms (blue) and binding site residue backbone atoms (red) as a function of time in MD simulations. All 6 systems reached equilibration state after 100ns with only little fluctuation (within 1Å) in the monitored RMSDs.

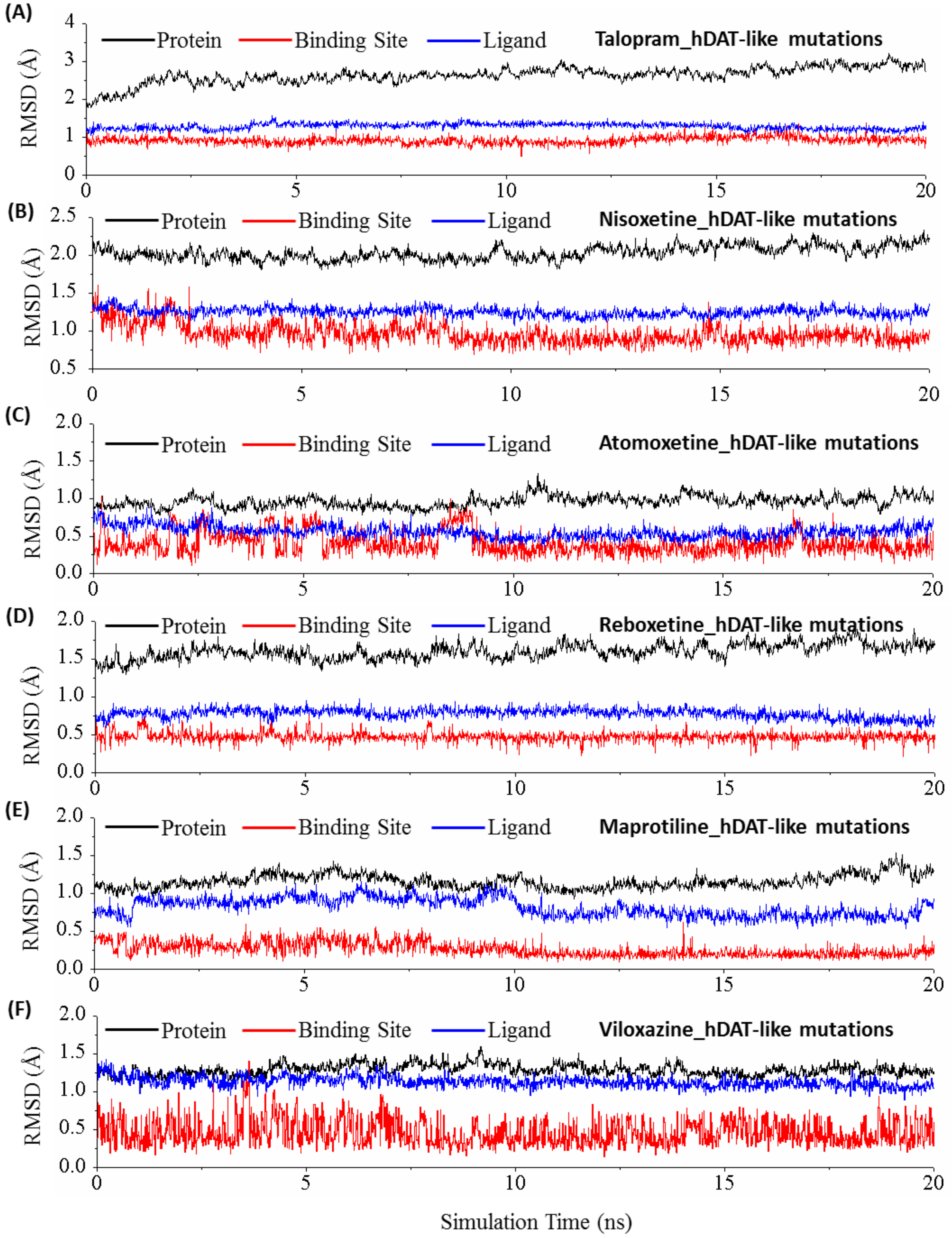

Figure S7. RMSDs of protein backbone atoms (black), ligand heavy atoms (blue) and binding site residue backbone atoms (red) of 6 studied sNRIs in complex with the hDAT-like hNET[32] as a function of time in MD simulations.

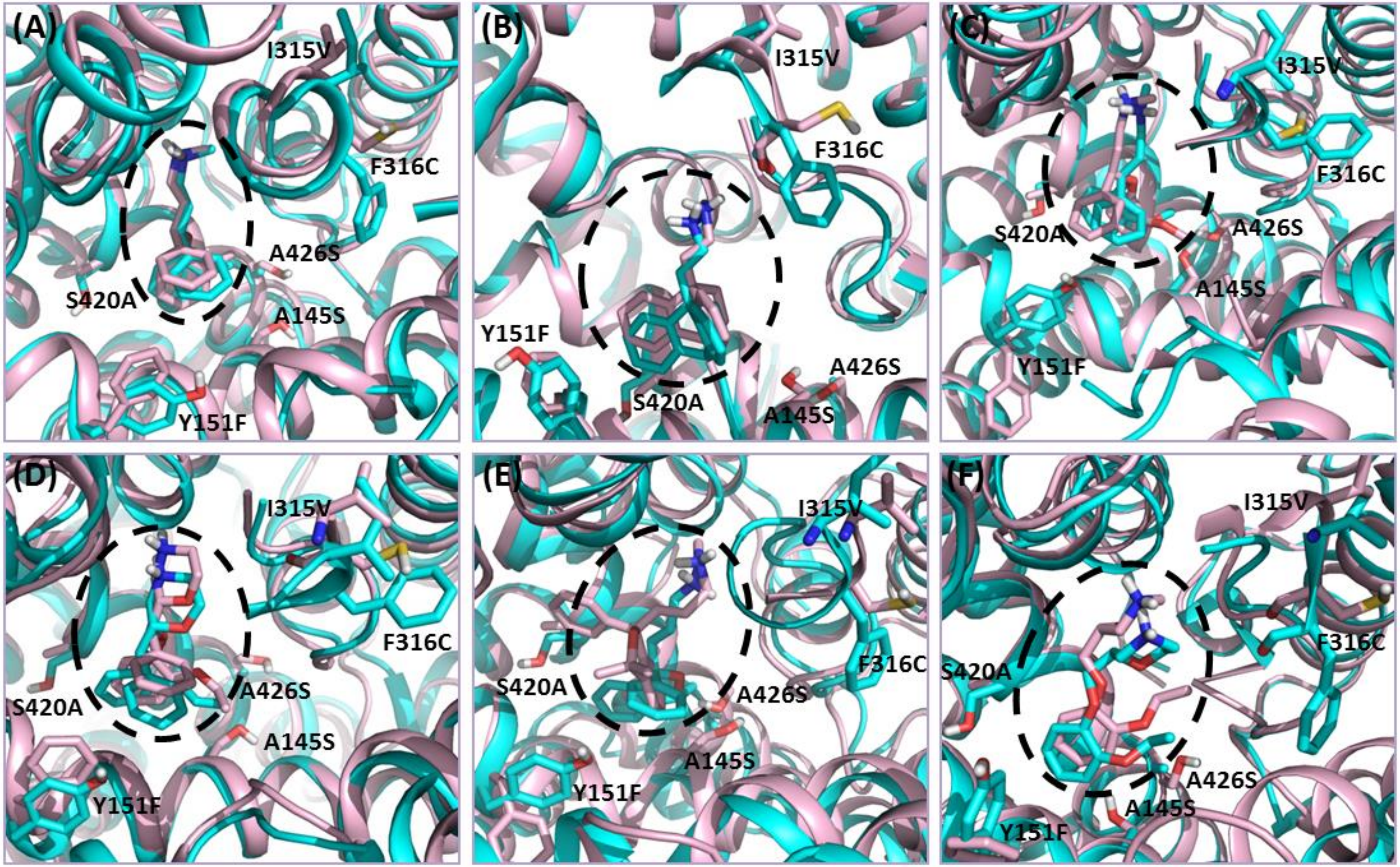

Figure S8. Structural superimposition of (A) atomoxetine, (B) maprotiline, (C) nisoxetine, (D) talopram, (E) viloxazine and (F) (S, S)-reboxetine binding to the wild type and hDAT-like hNET[32], The mutant residues and sNRIs were shown as a stick representation in wild type (cyan) and mutant (light pink) models, and sNRIs in the pocket were highlighted by a black dash circle.

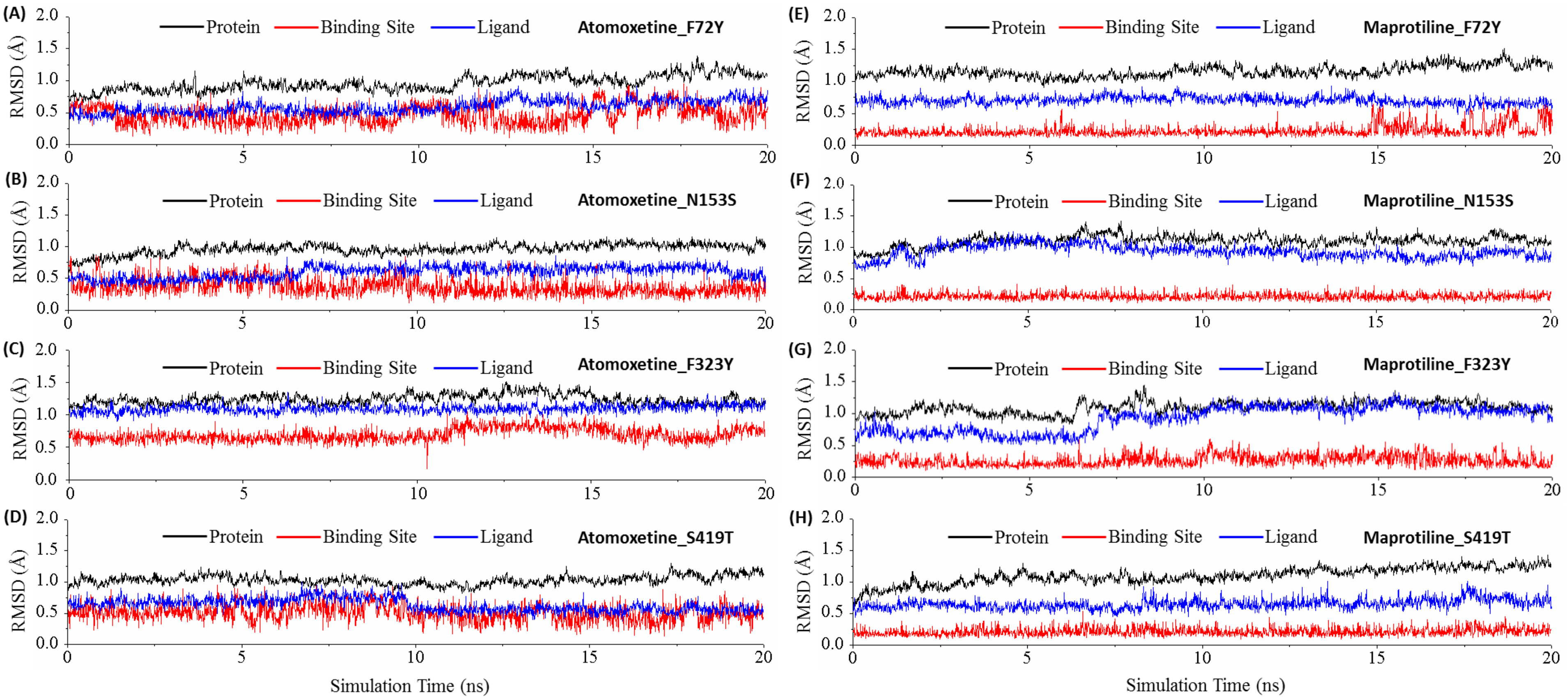

Figure S9. RMSDs of protein backbone atoms (black), ligand heavy atoms (blue) and binding site residue backbone atoms (red) of atomoxetine and maprotiline in complex with mutational hNET of 4 single-point mutations (S419T, F323Y, F72Y and N153S)[27] as a function of time in MD simulations.

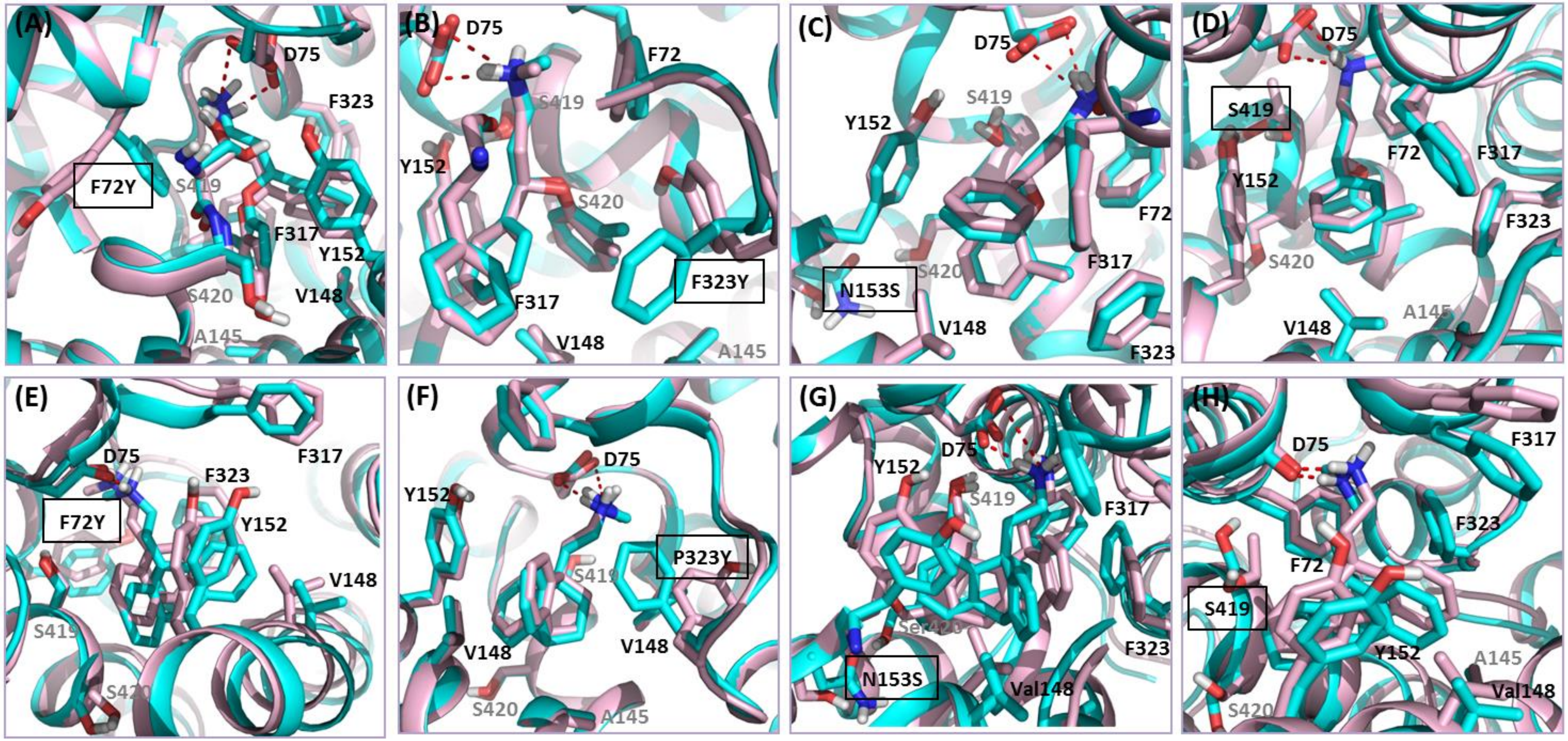

Figure S10. Structural superimposition of (A-D) atomoxetine and (E-H) maprotiline before and after single-point mutations (F72Y, N153S, F323Y and S419T) in hNET. Hot spot residues and sNRIs were shown as a stick representation in wild type (cyan) and mutant (light pink) models. The mutation residues were highlighted by a black square.

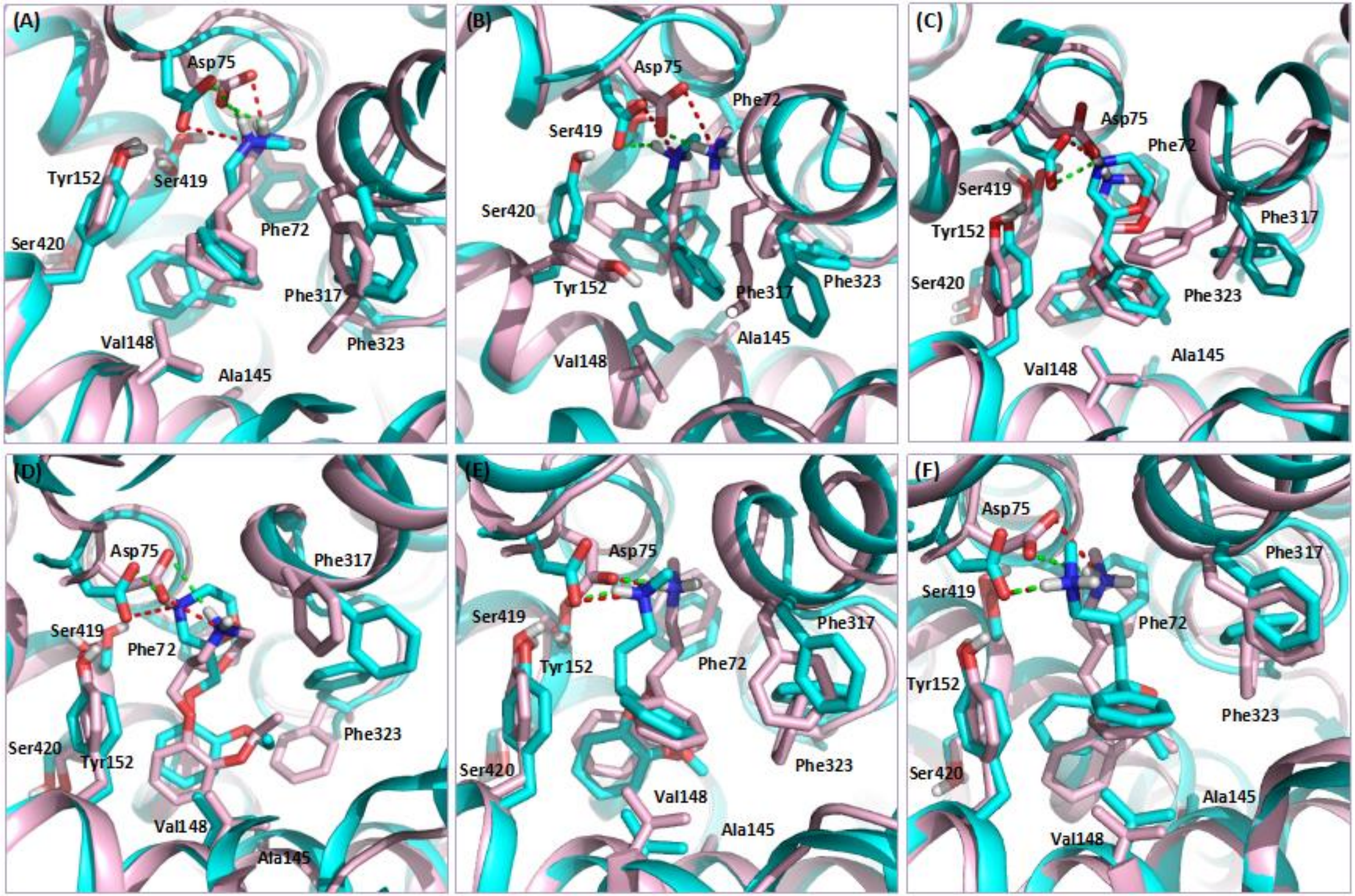

Figure S11. Structural alignment of the docked poses (cyan) and their corresponding representative snapshots (light pink) from MD simulation for 6 studied sNRIs in hNET. The salt bridge interaction was displayed in red dashed line and the hydrogen bond interaction was in green. (A) atomoxetine: the positively charged nitrogen and the attached hydrogen formed salt bridge and hydrogen bond with the carboxyl oxygen of Asp75 and carbonyl

oxygen of Phe72, respectively. Besides, two hydrophobic arms insert into the two hydrophobic pockets of the binding site with different orientations. The aromatic ring with o-methyl interacts with hydrophobic pocket consisted of the residues Phe72, Phe323, Ser419, Ser420 and Gly423, and other aromatic ring interacted with the hydrophobic residues Ala145, Val148, Gly149, Tyr152 and Phe317. (B) maprotiline: the polar nitrogen interacted with the carboxyl oxygen of Asp75 through the salt bridge. Meanwhile, the hydrogen attached to the positively charged nitrogen formed hydrogen bond with the carbonyl oxygen of Phe317. The bridge ring and two aromatic rings inserted into the hydrophobic region composed of by residues Ala145, Val148, Gly149, Tyr152, Phe323, Ser419 and Ser420. (C) (S, S)-reboxetine: the positively charged nitrogen and the attached hydrogen form salt bridge and hydrogen bond with the carboxyl oxygen of Asp75 and carbonyl oxygen of Phe72 respectively. The spatial configuration determined by two aromatic rings and morpholine ring made it less flexibility than atomoxetine. The aromatic ring without substituted group forms T-shaped π-π interactions with residues Tyr152 and Phe323 and π-π stacking interactions with Phe317. The aromatic ring with *o*-ethoxyl group interacts with residues Val148, Ser419, Ser420 and Gly423. (D) viloxazine: the morpholine ring provided the nitrogen, which was positively charged to form a salt bridge with the carboxyl oxygen of Asp75. The charged nitrogen forms cation-π interaction with Phe317 due to the charge-induced dipole. Meanwhile, the *o*-ethoxyl substituted aromatic ring was surrounded by the hydrophobic residues Val148, Ser419, Ser420 and Gly423, and formed T-shaped π-π interactions with the side chain of Tyr152. (E) nisoxetine: the positively charged nitrogen of amine formed salt bridge with the carboxyl of Asp75, meanwhile, the hydrogen attached to the charged nitrogen engages in a hydrogen bond with the oxygen of carbonyl. Two aromatic rings inserted into the hydrophobic pocket. The aromatic ring without substituted group interacts with the residue Ala145, Val148, Tyr152, Phe317 and Phe323 via hydrophobic interaction. The aromatic ring with the *o*-ethoxyl group mainly interacts with Ser419 and Ser420. (F) talopram: the salt bridge was formed between the carboxyl of Asp75 and the positively nitrogen of amine in the ligand. The two aromatic rings stretched into the hydrophobic pocket. The aromatic ring without substituted group mainly interacted with the Ala145, Val148, Ph317 and Phe323. Another aromatic ring interacted with the Tyr152 via π-π stack interaction. The Ser419, Ser420 and Phe72 interacted with the aromatic ring of spiro ring through hydrophobic interaction.

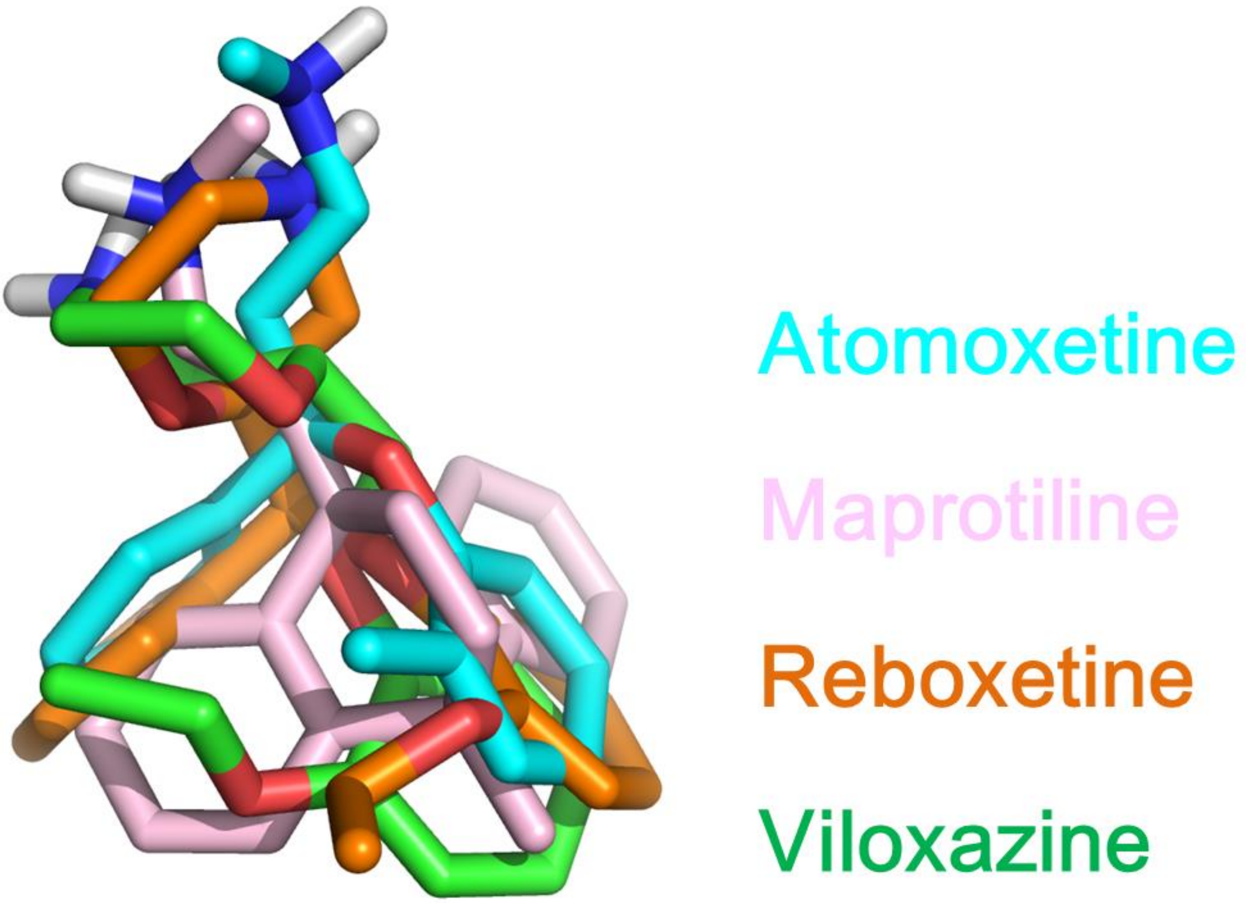

Figure S12. Superimposition of the approved sNRIs accommodating in the pock defined by 11 hot spot residues after MD simulations. Atomoxetine, maprotiline, reboxetine and viloxazine were represented in stick mode and colored by cyan, light pink, orange and green, respectively.

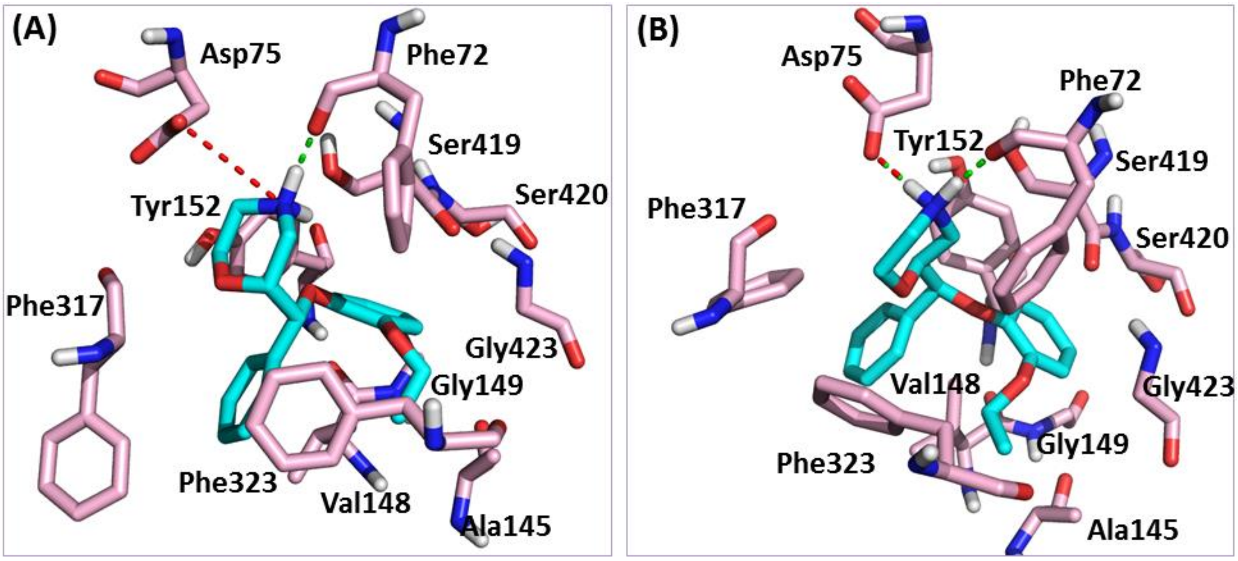

Figure S13. Binding mode (A) (R, R)-reboxetine and (B) (S, S)-reboxetine in the binding site of hNET. The reboxetine and binding site residues were shown in cyan and light pink, respectively. The salt bridge interaction was displayed in red dashed line and the hydrogen bond interaction was in green.

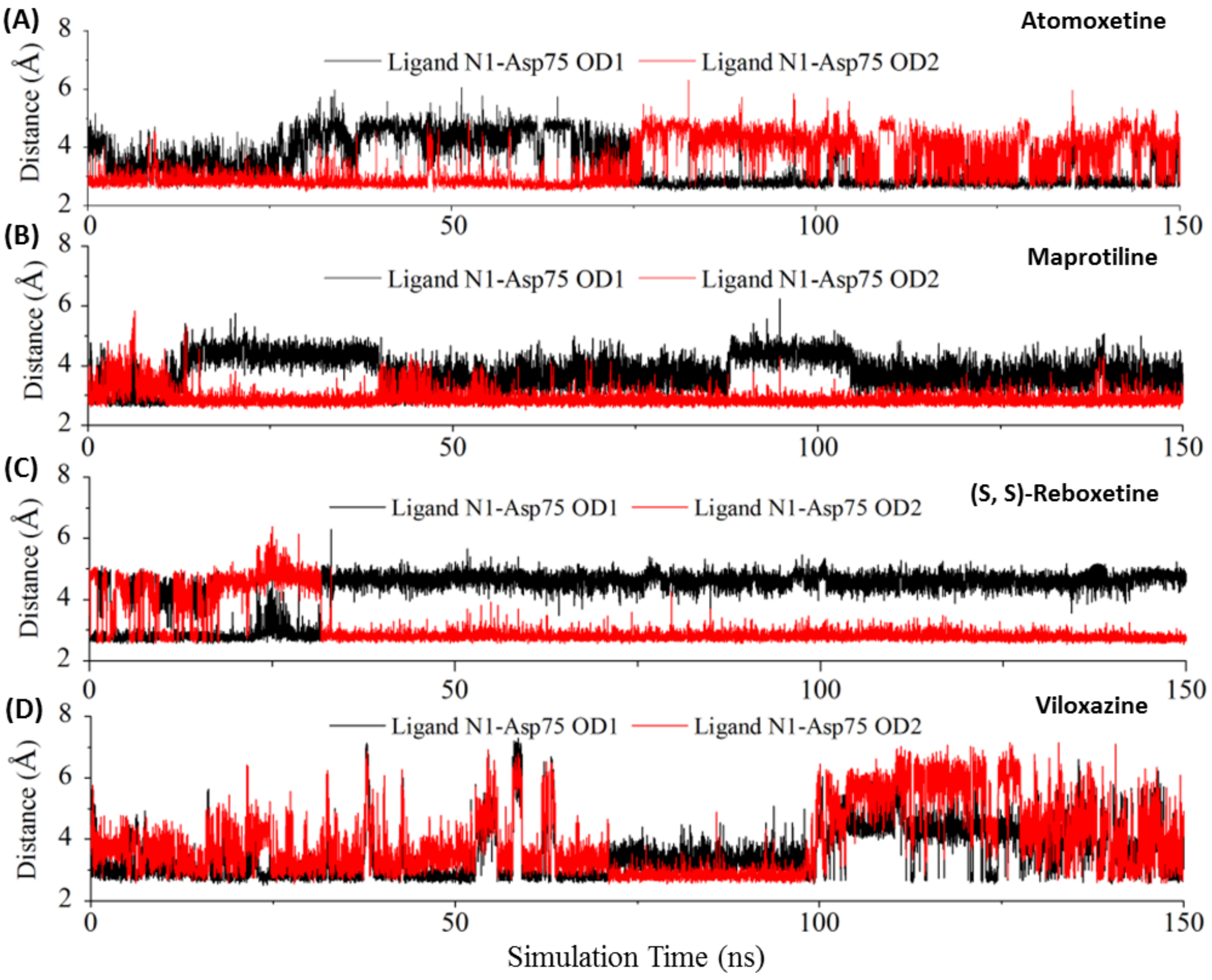

Figure S14. Distance between the ligands N1 and hNET's Asp75 OD in the approved sNRIs bound complexes during the 150ns MD simulation.

References

1. Glide v. 5.5, Schrödinger, LLC, New York, NY (2009). URL: http://www.schrodinger.com/
2. LigPrep v. 2.3, Schrödinger, LLC, New York, NY (2009). URL: http://www.schrodinger.com/
3. Kaminski, G. A., Friesner, R. A., Tirado-Rives, J. & Jorgensen, W. L. Evaluation and reparametrization of the OPLS-AA force field for proteins via comparison with accurate quantum chemical calculations on peptides. *J. Phys. Chem. B.* **105**, 6474-6487 (2001).
4. Epik v. 2.0, Schrödinger, LLC, New York, NY (2009). URL: http://www.schrodinger.com/
5. Penmatsa, A., Wang, K. H. & Gouaux, E. X-ray structures of Drosophila dopamine transporter in complex with nisoxetine and reboxetine. *Nat. Struct. Mol. Biol.* **22**, 506-508 (2015).
6. The PyMOL Molecular Graphics System v. 1.3, Schrödinger, LLC. URL: http://www.pymol.org/
7. Maestro v. 9.0, Schrödinger, LLC, New York, NY (2009). URL: http://www.schrodinger.com/
8. Lomize, M. A. *et al.* OPM database and PPM web server: resources for positioning of proteins in membranes. *Nucleic. Acids. Res.* **40**, D370-376 (2012).
9. Jo, S., *et al*. CHARMM-GUI Membrane Builder for mixed bilayers and its application to yeast membranes. *Biophys. J.* **97**, 50-58 (2009).
10. Jorgensen, W. L. *et al.* Comparison of simple potential functions for simulating liquid water. *J. Chem. Phys.* **79**, 926-935 (1983).
11. AMBER v. 14, University of California, San Francisco (2014). URL: http://ambermd.org/.
12. Hornak, V. *et al*. Comparison of multiple Amber force fields and development of improved protein backbone parameters. *Proteins* **65**, 712-725 (2006).
13. Dickson, C. J. *et al.* Lipid14: the amber lipid force field. *J. Chem. Theory Comput.* **10**, 865-879 (2014).
14. Joung, I. S. & Cheatham, T. E. Determination of alkali and halide monovalent ion parameters for use in explicitly solvated biomolecular simulations. *J. Phys. Chem. B* **112**, 9020-9041 (2008).
15. Wang, J. *et al.* Development and testing of a general amber force field. *J. Comput. Chem.* **25**, 1157-1174 (2004).
16. Bayly, C. I. *et al.* A well-behaved electrostatic potential based method using charge restraints for deriving atomic charges: the RESP model. *J. Phys. Chem.* **97**, 10269-10280 (1993).
17. Wang, J. *et al.* Automatic atom type and bond type perception in molecular mechanical calculations. *J. Mol. Graph. Model.* **25**, 247-260 (2006).
18. Gaussian 09 v. D.01, Gaussian, Inc., Wallingford CT (2009). URL: http://www.gaussian.com/index.htm/
19. Darden, T., York, D. & Pedersen, L. Particle mesh Ewald: An N·log(N) method for Ewald sums in large systems. *J. Chem. Phys.* **98**, 10089 (1993).
20. Humphrey, W., Dalike, A., and Schulten, K. VMD: visual molecular dynamics. *J. Mol. Graph.* **14**, 33-38 (1996).
21. Larini, L., Mannella, R. & Leporini, D. Langevin stabilization of molecular-dynamics simulations of polymers

by means of quasisymplectic algorithms. *J. Chem. Phys.* **126**, 104101 (2007).

22. Roe, D. R. & Cheatham, T. E. PTRAJ and CPPTRAJ: software for processing and analysis of molecular dynamics trajectory data. *J. Chem. Theory. Comput.* **9**, 3084-3095 (2013).

23. Kollman, P. A. *et al.* Calculating structures and free energies of complex molecules: combining molecular mechanics and continuum models. *Acc. Chem. Res.* **33**, 889-897 (2000).

24. Massova, I. & Kollman, P. Combined molecular mechanical and continuum solvent approach (MM-PBSA/GBSA) to predict ligand binding. *Perspect. Drug. Discov. Des.* **18**, 113-135 (2000).

25. Onufriev, A. B., D.Case, D. A. Exploring protein native states and large-scale conformational changes with a modified generalized born model. *Proteins* **55**, 383-394 (2004).

26. Weiser, J., Shenkin, P. S. & Still, W. C. Approximate atomic surfaces from linear combinations of pairwise overlaps (LCPO). *J. Comput. Chem.* **20**, 217-230 (1999).

27. Sorensen, L. *et al.* Interaction of antidepressants with the serotonin and norepinephrine transporters: mutational studies of the S1 substrate binding pocket. *J. Biol. Chem.* **287**, 43694-43707 (2012).

28. Bymaster, F. P. *et al*. Atomoxetine increases extracellular levels of norepinephrine and dopamine in prefrontal cortex of rat: a potential mechanism for efficacy in attention deficit/hyperactivity disorder. *Neuropsychopharmacology* **27**, 699-711 (2002).

29. Tatsumi, M. *et al.* Pharmacological profile of antidepressants and related compounds at human monoamine transporters. *Eur. J. Pharmacol.* **340**, 249-258 (1997).

30. Kung, H. F. *et al*. 2-(2-(dimethylaminomethyl)phenoxy)-5-iodophenylamine: an improved serotonin transporter imaging agent. *J. Med. Chem.* **47**, 5258-5264 (2004).

31. Andersen, J. *et al.* Molecular determinants for selective recognition of antidepressants in the human serotonin and norepinephrine transporters. *Proc. Natl. Acad. Sci. USA* **108**, 12137-12142 (2011).

32. Benson, N. *et al.* Estimation of binding rate constants using a simultaneous mixed-effects method: application to monoamine transporter reuptake inhibitor reboxetine. *Br. J. Pharmacol.* **160**, 389-398 (2010).

33. Andersen, J. *et al*. Binding site residues control inhibitor selectivity in the human norepinephrine transporter but not in the human dopamine transporter. *Sci. Rep.* **5**, 15650 (2015).
